# Caesarean section Robson classification, complications, and lessons learned in a rural hospital in Walikale, North Kivu, Democratic Republic of Congo: a cross-sectional study

**DOI:** 10.1016/j.xagr.2025.100586

**Published:** 2025-11-23

**Authors:** Geoges Lubuto Bushu, Christophe Kambale, Seraphin Kikwabantu, Faida Boelongo Benedite, Wolfgang Weber, Benjamin O. Black, Kim J.C. Verschueren

**Affiliations:** 1 Department of Obstetrics, General Referral Hospital Walikale, Walikale, North Kivu, Democratic Republic of the Congo (Bushu, Kambale, Kikwabantu, Benedite, and Verschueren); 2 Médecins Sans Frontières, Project Walikale, North Kivu (Quartier Les Volcans, Goma, Nord Kivu, DR Congo) of Operational Centre Amsterdam, Amsterdam, The Netherlands (Kambale, Benedite, Weber, Black, and Verschueren); 3 Department of Obstetrics, Wilhelmina Children’s Hospital, University Medical Centre of Utrecht, University of Utrecht, Utrecht, The Netherlands (Verschueren)

**Keywords:** audit, caesarean section, complications, indications, Robson

## Abstract

**Background:**

Globally rising caesarean section (CS) rates have resulted in more women with a scarred uterus. In low-resource, high-fertility settings, this likely contributes to a growing number of high-risk pregnancies and births. Exploring and understanding this trend in low-resource settings is essential to reduce maternal and perinatal mortality globally.

**Objective:**

This study aimed to assess CS practices in Walikale, eastern Democratic Republic of Congo (DRC), using the Robson classification, clinical indications, maternal and perinatal complications, and quality of care, to improve CS decision-making and clinical outcomes.

**Study Design:**

A cross-sectional study was conducted at the General Referral Hospital of Walikale, North Kivu, DRC, from January 1 to March 31, 2024. Data from all births were reviewed, and clinical case reviews were performed systematically for each CS by a team of health care providers. Descriptive statistics were used for analysis.

**Results:**

The CS rate was 15.7%, with 136 CS of 868 births. Women in Robson group 5 (previous CS, term, cephalic presentation), accounted for 50% of all CS (68/136). Among CS in Robson groups 6 to 10 (malpresentation, twins, preterm), 65.6% (21/32) were also performed in women with a previous CS. Vaginal birth after CS (VBAC) rates were high: 71.2% (79/111) after one and 50.0% (23/46) after two previous CS. Among women with a previous CS (191/868, 22.0% of the population), uterine rupture occurred in 5.8% (11/191) and abnormally invasive placenta in 3.7% (7/191). Surgical site infections occurred in 7.4% (10/136) of CS. Perinatal mortality was 60 per 1000 births (53/886 total births). Case reviews showed that 9.6% (13/136) of CS were performed “too late” and 24.3% (33/136) “too soon.” Positive findings included high antenatal care attendance and short decision-to-CS intervals. Recommendations following from case review included strengthening clinical supervision and training health workers, team decision-making for CS with relative indications, ensuring access to the hospital, medication, blood, and contraception, especially for women with prior CS.

**Conclusion:**

In this conflict-affected, high-fertility, low-resource context, the CS rate is rising as well as the proportion of women with a scarred uterus. The majority of CS are performed in women with a previous CS. VBAC rates are notably high, though both VBAC and multiple repeat CS carry important safety considerations. Applying the Robson classification is challenging in low-resource settings, where gestational age is often unknown. To optimize CS care and reduce adverse outcomes, it is crucial to prevent the first unnecessary CS, ensure timely and appropriate indications through context-adapted guidelines, clinical supervision, and routine case reviews, and strengthen both basic and comprehensive antenatal and obstetric care.

**AJOG Global Reports at a Glance**

**Why was this study conducted?**

In this conflict-affected, high-fertility, low-resource setting, the caesarean section (CS) rate is rising, with 22% of women in labor presenting with a uterine scar, and most CS performed in those with multiple previous CS.

**Key findings**

Despite achieving the highest reported VBAC rate in such settings, maternal and perinatal complication rates remain high.

**What does this add to what is known?**

To improve outcomes, it is crucial to prevent the first unnecessary CS, enhance VBAC safety, strengthen clinical oversight, and routinely review CS indications.

## Introduction

Achieving Sustainable Development Goal 3, which aims for a global maternal mortality ratio (MMR) below 70 per 100,000 live births by 2030 with no country exceeding an MMR above 140 by 2030, presents a challenge for many countries, particularly those affected by humanitarian crises, conflict, and disasters.^1^ The eastern Democratic Republic of Congo (DRC) has endured decades of armed conflict, poor infrastructure, extreme poverty and a disrupted healthcare system, hindering further progress. The DRC, ranked among the nine most fragile countries in 2020, has an MMR of 547, with a 15-year-old girl’s lifetime risk of dying from maternal causes at 1 in 29 (500 times higher than in high-income countries).^2^ DRC accounts for 7.5% of maternal deaths globally, with 22,000 maternal deaths per year, ranking third after Nigeria (82,000) and India (24,000).^3^

Universal access to caesarean section (CS) is crucial for improving maternal and perinatal outcomes.^4^, ^5^, ^6^ Over the past decades, Médecins Sans Frontières (MSF) has provided humanitarian medical care, including comprehensive obstetric and newborn care to regions in DRC with the poorest access to care and the highest mortality burden.^7^ While addressing the unmet need for CS, it is equally important to recognize that overuse often coexists with underuse.^8^ Unnecessary CS not only strains resources but also increases maternal and perinatal mortality, particularly in low-resource settings, where the risk of death from CS is 100 times higher (10 per 1000 CS) than in high-income countries.^9^ Thus, efforts to expand CS access must be accompanied by improvements in decision-making and the quality of peri- and postoperative care.^4^^,^^6^

The World Health Organization (WHO) and other global policy makers emphasize the need for more research to better understand CS use.^5^^,^^10^, ^11^, ^12^, ^13^, ^14^ The Robson classification system, introduced in 2011 and adopted by over 50 countries and 200 health facilities, is the global standard for assessing, monitoring and comparing CS rates across health facilities and trends over time.^15^, ^16^, ^17^ Although several small-scale studies in DRC have used the Robson classification, more comprehensive data, including reasons for CS and associated complications, are needed to fully understand the challenges, optimize CS use and ultimately reduce maternal and perinatal mortality.^14^^,^^18^, ^19^, ^20^, ^21^

The primary objective of this study is, therefore, to assess CS according to the Robson classification, determine the primary clinical indications, and evaluate maternal and perinatal complications in a rural referral hospital in Walikale, North Kivu, DRC. The secondary objective is to assess obstetric quality of care through clinical reviews of CS and provide detailed, context-specific recommendations to improve maternal and perinatal outcomes.

## Materials and methods

### Study design

We conducted a cross-sectional study of all births and an analysis of all CS in the General Referral Hospital (l’Hôpital Général de Référence, HGR) of Walikale for 3 months, from January 1 to March 31, 2024.

### Study setting

Walikale town is in the Walikale Health Zone, located in North Kivu province, eastern DRC. While there had been a relative decline in conflict intensity and some improvements in the humanitarian situation in recent years; in 2025 (poststudy) Walikale saw an escalation in violence with M23 rebel groups seizing control of the area.^22^

MSF has been providing medical, technical, and financial support to the Walikale Health Zone since 2012 for primary and secondary care for a population with a high disease burden.^7^ Walikale Health Zone has a population of approximately 200,000 inhabitants. The Ministry of Health operates HGR Walikale along with 18 rural health centers, several of which receive partial support from MSF. HGR is a 200-bed facility in Walikale staffed by 12 generalist doctors, 10 nurse-midwives, and no medical specialists. There are approximately 4000 hospital births per year.^7^ There is no Intensive Care Unit. Blood for transfusion is mostly sourced from Goma, the provincial capital, and must be flown in by helicopter. A maternity waiting home is available for women who live far from the hospital and/or have high-risk pregnancies.^23^ Antenatal care (ANC) is provided in health centers and is free of charge through government support, although chronic shortages of medication and vaccines result in sub-optimal care and out-of-pocket expenses. There is no ambulance care, and women and their families arrange transport themselves to the hospital, typically by motorcycle.

### Participants and data collection

This study included all women who gave birth in HGR, Walikale, from January 1 to March 31, 2024, with a minimum gestational age of 28 weeks and/or 1000 grams, to adhere to WHO guidelines and viability in our setting.^24^^,^^25^ Data were collected from hospital admission registers, birth registers, and discharge registers (for both mother and baby). Data were entered into a password-protected Microsoft Excel file (version 16.87, 2024), ensuring that all personal information was omitted. Medical records of all births were collected directly after discharge, although data were entered into the database at least 6 weeks postpartum to ensure complications were captured. Robson classification was conducted by two of the authors manually, and cross-checked with an Excel formula, classifying each case into its Robson group *(*Additional File 1*, an overview of Robson classification).*^15^ For all women who gave birth by CS, additional data on the procedure (eg, clinical CS indication, phase of labor, type of skin incision) were extracted and a case summary was made by two of the authors.

CS case reviews were conducted by a five-member study team (all are coauthors) with extensive clinical experience in low-resource obstetric settings, including one senior obstetrician, one senior medical doctor, two midwife supervisors, and one midwife. Any member who had been clinically involved in a particular case was excluded from its review (four cases). Each CS was summarized and independently reviewed in a blinded manner, followed by a structured group discussion to reach consensus.

Although no universally accepted standardized CS audit tool exists, we developed based on clinical relevance, MSF protocols, and previously published studies auditing CS.^26^^,^^27^ The assessment focused particularly on the timing of CS in relation to MSF protocol recommendations. An example of a CS considered performed “*too soon*” (and thus unnecessary) is if the indication was “no progress,” but membranes were still intact and/or oxytocin augmentation had not been considered. Conversely, a CS was considered “*too late*” when, for instance, a woman with three or more previous caesarean scars only underwent surgery after entering active labor. The assessment also focused on the urgency of the CS (planned, unplanned, or emergency) and the strength of the clinical indication, classified as absolute or relative according to MSF protocols.^26^ Conditions such as placenta previa, suspected uterine rupture, or history of >3 previous CS were considered absolute CS indication, whereas fetal distress or poor progress were categorized as relative indications. The case review concluded with identified strengths and case-specific recommendations. See Additional File 2 for an extensive elaboration of all the variables and definitions used for clinical case review*.* Despite the subjectivity, consistency was assumed as the same audit team reviewed all cases. In case of disagreement during the group discussion, the opinion of an independent senior obstetrician-gynecologist, outside of Walikale, was sought. Findings from these clinical case reviews were added to the study database.

### Variables and definitions

A detailed overview of all variables, along with their definitions and categorizations, which align with international standards and literature, is provided in Additional File 2. Variables included maternal characteristics, general and obstetric history, ANC, pregnancy and childbirth outcomes, maternal complications (including maternal deaths and near-misses, up to 42 days postpartum—according to WHO near-miss tool), and perinatal outcomes (including perinatal deaths and neonatal near-misses, up to 28 days postbirth). Classification of births into one of the 10 Robson Groups was conducted using the following variables: parity (primiparous/multiparous), fetal number (singleton/multiple), fetal presentation (cephalic/breech/transverse), onset of labor (spontaneous/induction), previous CS (yes/no), and gestational age (term/preterm).^15^ Robson Group 5 was subdivided for clinical interpretation: R5.1 included women with one previous CS, R5.2 with two previous CS, and R5.3 with >3 previous CS.

In cases where gestational age was unknown, the distinction between term and preterm was based on the duration of amenorrhea (less than 9 months considered preterm), birth weight (less than 2500 grams considered preterm), and/or clinical assessment of newborn maturity. Babies were classified as preterm if they showed characteristic features such as lean body habitus, thin translucent skin, abundant lanugo, low muscle tone, and less developed ear cartilage or genitalia. Clinical case reviews included variables such as the leading clinical indication (ie, principal, most important reason), phase and stage of labor, decision-to-delivery interval (minutes), duration of the procedure (minutes), anaesthesia and incision techniques, complications, informed consent, and documentation. They also included the following qualitative factors: (1) absolute vs relative indication for CS (based on the MSF guideline, in which maternal life-threatening complications are absolute indications), (2) quality of decision-making (too late, too soon, good moment), (3) urgency level, (4) positive practices and (5) recommendations^26^ (Additional File 2). The latter two were reported using the Three Delays Model, a framework examining delays related to patient/community factors (first delay), accessibility and efficiency of referral (second delay), and quality of care at facility level (third delay).^28^

### Analysis

Study data were analyzed using IBM SPSS Statistics (version 29.0.2, 2024) and R. Descriptive statistics were applied to summarize the variables. Prevalence estimates were reported with 95% confidence intervals, calculated using the Wilson method to improve accuracy for smaller sample sizes and proportions near 0 or 1. Comparisons between vaginal birth and CS were performed using Chi-square tests for categorical variables with expected cell counts >10 and Fisher’s exact test for smaller samples (*n*<10), with *P* values reported accordingly.

Missing data were excluded from comparative analyses. Data imputation for variables such as HIV status and ANC visits was not performed, as these data were not missing at random and imputation would likely introduce bias. Moreover, no additional analyses were performed as it would not impact any statistical model. Missing gestational age values were pragmatically estimated using clinical parameters, resulting in no missing cases for Robson classification. Because the analysis included all births during the 3-month study period, no formal sample size or power calculations were required. Multivariate regression was not undertaken, as the study aimed to provide a descriptive overview of CS rates, indications, and outcomes rather than to identify independent risk factors.

## Results

### Study population

**Figure 1:**
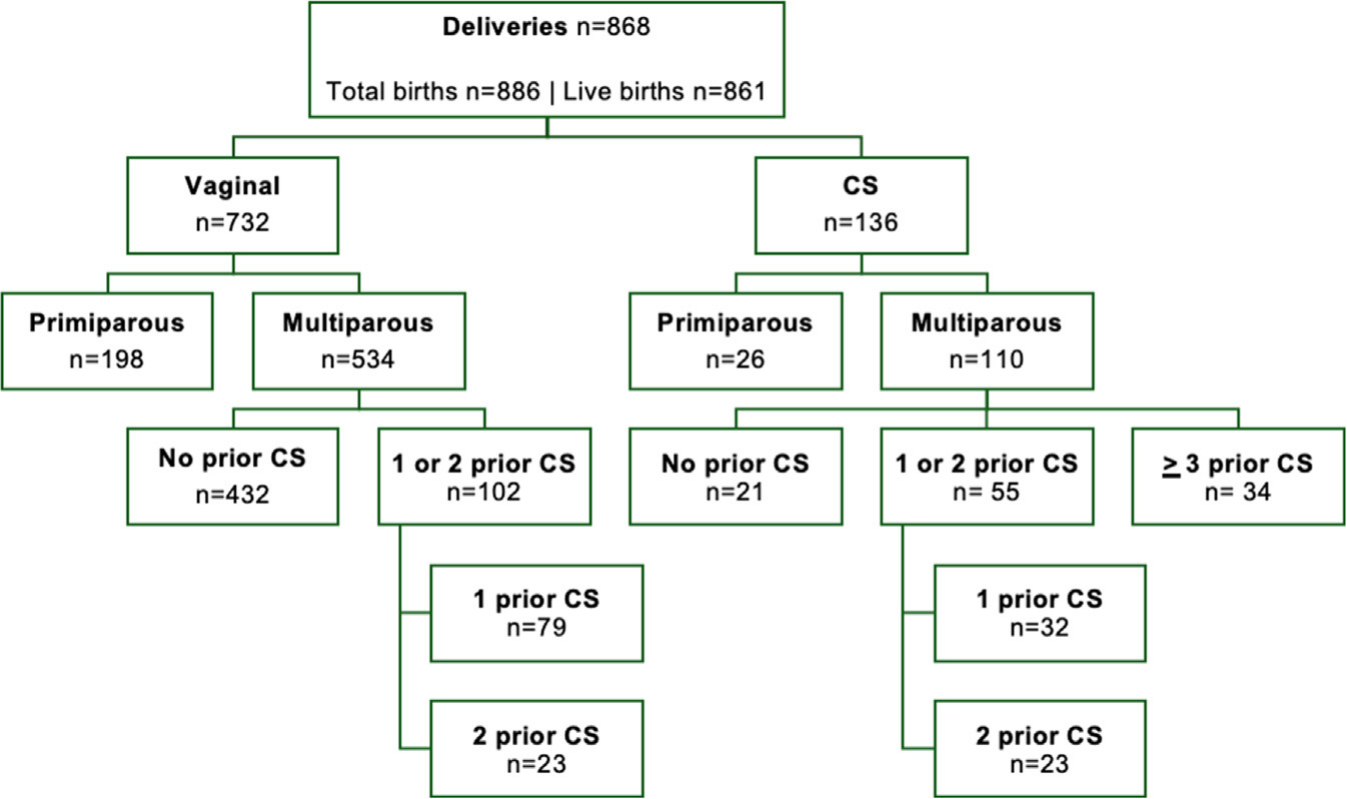
Flowchart of the study population

A total of 868 women gave birth in HGR Walikale during the 3-month study period in 2024, resulting in 886 babies, including 16 sets of twins and 1 set of triplets. Of these, 861 (97.1%) were live births. There were 136 CS, yielding a hospital CS rate of 15.7% (95% CI 13.4%–18.2%). Figure 1 presents the study population by mode of birth, parity, and previous CS.

Bushu & Kambale et al. Caesarean section Robson classification, complications, and lessons learned. AJOG Glob Rep 2025.

### Maternal and perinatal characteristics

Table 1 presents maternal and perinatal characteristics, with a more detailed version available in Additional File 3. Of all women giving birth, 191 (22.0%, 95% CI 19.4%–25.0%) had one or more previous CS scars, of which 20 (10.5%) had four or more previous CS. The vaginal birth rate after one prior CS (vaginal birth after CS [VBAC]-1) was 71.2% (*n*=79/111, 95% CI 62.1%–78.8%), and after two prior CS (VBAC-2), 50.0% (*n*=23/46, 95% CI 36.1%–63.9%). For one in five women, the HIV status was unknown (*n*=174), either due to unavailability of the test or because testing was declined. Vacuum-assisted birth was attempted in 52 (6.0%) of births, with 42 (80.8%) resulting in successful vaginal birth. Tubal ligation was performed in 5 (11.9%) of 42 great grand multiparous women (P10 and higher) and in 9 (15.8%) of 57 women with >3 previous CS. Gestational age was unknown in 421 women (48.5%) due to an unknown last menstrual period and/or the absence of an ANC visit in the first trimester. Among those with known gestational age, preterm birth rate was 8.3% (*n*=37/447) and post-term delivery rate was 13.0% (*n*=58/447). Low birth weight was recorded in 147 (16.9%), with no neonatal survival <1500 grams (*n*=0/10). Neonatal asphyxia occurred in 51 of 840 babies born alive (6.1%), with a higher rate among babies born by CS (17.2%, *n*=22/128).

**Table 1:** Maternal and perinatal characteristics of all births in Walikale

	Total *n*=868	Vaginal birth *n*=732 (%)	CS *n*=136 (%)	*P* value
**Maternal and pregnancy characteristics**				
**Maternal age** (y)				
<20	200 (23.0)	178 (24.3)	22 (16.2)	.11
20–34	585 (67.4)	484 (66.1)	101 (74.3)	
≥35	83 (9.6)	70 (9.6)	13 (9.6)	
**Distance of village (min)**				
<30 min	523 (60.5)	465 (63.8)	58 (42.6)	<.01
≥30 and <120 min	230 (26.6)	174 (23.9)	56 (41.2)	
≥120 min	112 (12.9)	90 (12.3)	22 (16.2)	
*Missings*	*3 (0.3)*	*3 (0.4)*	*0 (0)*	
**Parity**				
Primiparity	224 (25.8)	198 (27.0)	26 (19.1)	.19
Low multiparity (P1–5)	409 (47.2)	339 (46.3)	70 (51.5)	
Grand multiparity (P6–9)	193 (22.2)	158 (21.6)	35 (25.7)	
Grand grand multipara (*P*≥10)	42 (4.8)	37 (5.1)	5 (3.7)	
**Previous CS**				
Yes	191 (22.0)	102 (13.9)	89 (65.4)	<.01
No	677 (78.0)	630 (86.1)	47 (34.6)	
**Number of previous CS**				
One	111 (12.8)	79 (10.8)	32 (23.5)	<.01
Two	46 (5.3)	23 (3.1)	23 (16.9)	
Three or more	34 (3.9)	-	34 (25.0)	
**Antenatal care**				
Yes	765 (99.4)	649 (99.5)	116 (98.3)	.31
No	5 (0.6)	3 (0.5)	2 (1.7)	
*Missings*	*98 (11.3)*	*80 (10.9)*	*18 (13.2)*	
**HIV**				
Positive	11 (1.6)	11 (1.9)	0 (0)	.39
Negative	683 (98.4)	577 (98.1)	106 (100)	
*Missings*	*174 (20.0)*	*144 (19.7)*	*30 (22.1)*	
**Traditional medication used**	35 (4.0)	29 (4.0)	6 (4.4)	.81
**Number of fetuses**				
Singleton	851 (98.0)	724 (98.9)	127 (93.4)	<.01
Twins	16 (1.9)	7 (1.0)	9 (6.6)	
Triplets	1 (0.1)	1 (0.1)	0 (0)	
**Fetal presentation**				
Cephalic	840 (96.8)	718 (98.1)	122 (89.7)	<.01
Breech	21 (2.4)	14 (1.9)	7 (5.1)	
Transverse	7 (0.8)	0 (0)	7 (5.1)	
**Childbirth information**				
**Gestational age**				
Very preterm (<32 wk)	7 (1.6)	4 (1.1)	3 (3.9)	.72
Moderate preterm (32–33^+6^ wk)	10 (2.2)	8 (2.2)	2 (2.6)	
Late preterm (34–36^+6^ wk)	20 (4.5)	16 (4.3)	4 (5.2)	
Term (37–41^+6^ wk)	352 (78.7)	293 (79.2)	59 (76.6)	
Post-term (≥42 wk)	58 (13.0)	49 (13.2)	9 (11.7)	
*Missings*	*421 (48.5)*	*362 (49.5)*	*59 (43.2)*	
**Labor induction**	17 (2.0)	12 (1.6)	5 (3.7)	.12
**Oxytocin augmentation**	35 (4.0)	26 (3.6)	9 (6.6)	.10
**Episiotomy**	111 (12.7)	107 (14.6)	4 (2.9)	<.01
**Vacuum-assisted delivery attempted**	52 (6.0)	42 (5.7)	10 (7.4)	.29
**Contraception by tubal ligation**	18 (2.1)	6 (0.8)	12 (8.8)	<.01
**Perinatal characteristics**				
**Birth weight**				
Very low (<1500 grams)	5 (1.2)	5 (0.7)	5 (3.7)	<.01
Low (1500–2499 grams)	137 (15.8)	111 (15.2)	26 (19.1)	
Normal (≥2500 grams)	720 (83.2)	615 (84.1)	105 (77.2)	
*Missings*	*1 (0.1)*	*1 (0.1)*	*0 (0)*	
**Apgar 5 min** (*n*=840, excluding stillbirths)				
Asphyxia (score 1–6)	51 (6.1)	29 (4.1)	22 (17.2)	<.01
Difficulty adapting (score 7–8)	297 (35.6)	237 (33.5)	60 (46.9)	
Normal (score 9–10)	487 (58.3)	441 (62.4)	46 (35.9)	
*Missings*	*5 (0.6)*	*5 (0.7)*	*0 (0)*	
**Admission to neonatal unit**	80 (9.2)	44 (6.0)	38 (27.9)	<.01

### Robson classification

Table 2 presents the Robson classification. The largest contribution to the overall CS rate was Group 5 (ie, multiparous women with at least one previous CS scar and a single cephalic pregnancy of ≥37 weeks’ gestation), accounting for 50.0% (*n*=68/136) of all CS, followed by Group 1, accounting for 14.0% (*n*=19/136) of all CS. While Group 5 had the largest contribution to the overall CS rate, the CS rate within the group was 41.5% (*n*=68/164). Within this group, 39.7% (*n*=27/68) were not offered VBAC as they had >3 previous CS.

**Table 2:** Robson classification of all births in Walikale

	No. of CS	No. of deliveries	Group size^a^ (%)	CS rate^b^ (%)	Absolute contribution^c^ (%)	Relative contribution^d^ (%)
**Robson 1** Nulliparous, with a single cephalic pregnancy, at ≥37 wk’ gestation, spontaneous labor.	19	198	22.8	9.6	2.1	14.0
**Robson 2** Nulliparous, with a single cephalic pregnancy, at ≥37 wk’ gestation, induced labor or CS before labor.	2	4	0.5	50.0	0.2	1.5
**Robson 3** Multiparous, without a previous CS scar, with a single cephalic pregnancy at ≥37 wk’ gestation, spontaneous labor.	12	409	47.1	2.9	1.4	8.8
**Robson 4** Multiparous, without a previous CS scar, a single cephalic pregnancy, at ≥37 wk’ gestation, induced labor or CS before labor.	1	8	0.9	12.5	0.1	0.7
**Robson 5** Multiparous, with at least one CS scar and a single cephalic pregnancy, at ≥37 wk’ gestation.	68	164	18.9	41.5	7.8	50.0
**5.1—**One previous CS	22	97	11.1	22.7	2.5	16.2
**5.2—**Two previous CS	19	40	4.6	47.5	2.2	14.0
**5.3—**Three or more previous CS	27	27	3.1	100	3.1	19.9
**Robson 6** Nulliparous, with a single breech pregnancy.	1	4	0.5	25	0.1	0.7
**Robson 7** Multiparous, with a single breech pregnancy (incl. previous CS scar).	4	14	1.6	28.6	0.5	2.9
**Robson 8** Multiple pregnancies (incl. previous CS scar).	9	17	1.9	52.9	1.0	6.6
**Robson 9** Single pregnancy with transverse or oblique lie (incl. previous CS scar).	8	8	0.9	100	0.9	5.8
**Robson 10** Single cephalic pregnancy at ≤36 wk’ gestation (incl. previous CS scar).	12	42	4.8	28.6	1.4	8.8
**Total**	136	868	N/A	15.7	15.7	100%

a Group size = number of deliveries in group/total number of deliveries

b CS rate per group = number of CS in group/number of deliveries in group

c Absolute contribution = number of CS in group/total number of deliveries

d Relative contribution = number of CS in group/total number of CS.

### Clinical CS indication

**Figure 2:**
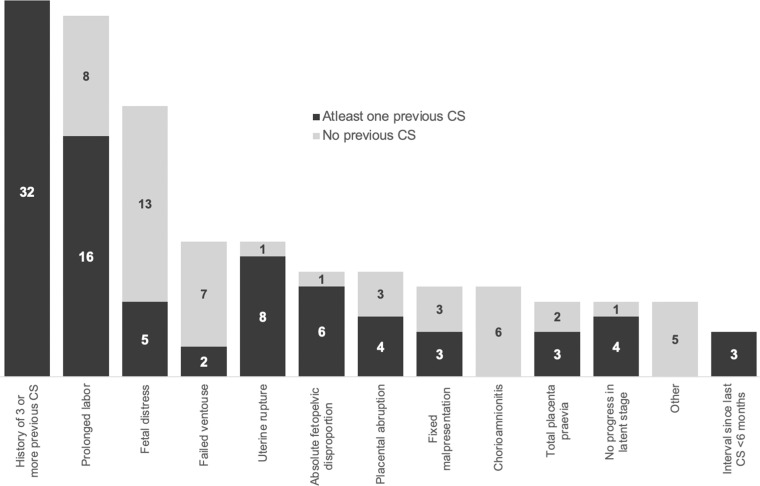
Leading clinical CS indications in Walikale with and without prior CS

**Figure 3:**
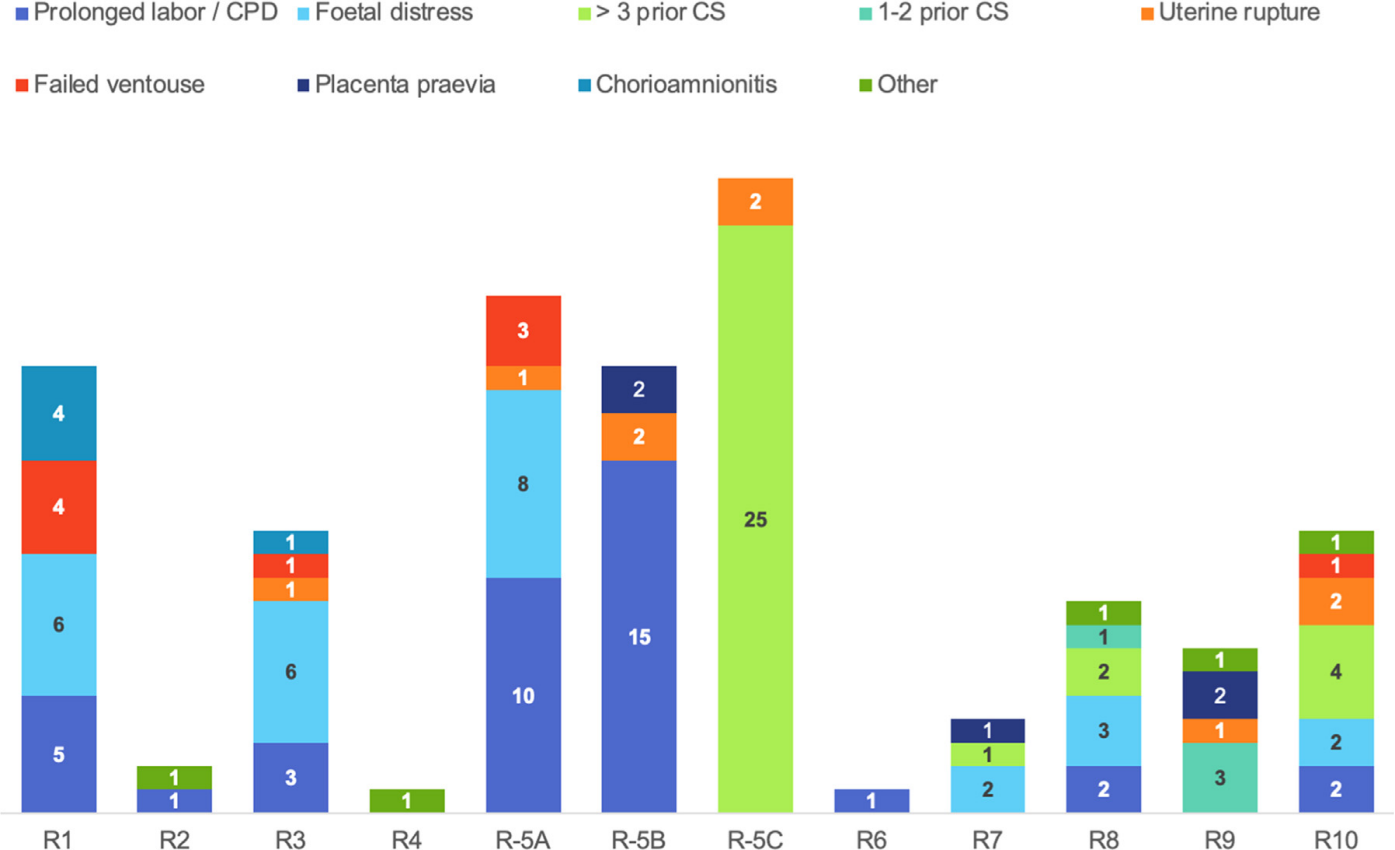
Robson-group-based leading clinical CS indications in Walikale

Figure 2 depicts the leading clinical CS indications, that is, the primary reason for CS. The most common principal indication for CS was a history of >3 previous CS with 32/136 (23.5%). This was followed by *prolonged labor* in 24/136 (17.6%), of whom 16 women had one or two previous CS scars. Fetal distress was the third leading CS indication, contributing to 18/136 (13.2%) of all CS. Figure 3 depicts the leading clinical CS indications per Robson-group. The majority of CS in Robson group 5A (one previous CS) and 5B (two previous CS) were performed for prolonged labor.

Bushu & Kambale et al. Caesarean section Robson classification, complications, and lessons learned. AJOG Glob Rep 2025.

Bushu & Kambale et al. Caesarean section Robson classification, complications, and lessons learned. AJOG Glob Rep 2025.

### Maternal and perinatal outcomes

Table 3 presents maternal and perinatal outcomes, with a more detailed version available in Additional File 3. Maternal and perinatal complications per Robson group are found in Additional File 4. There were four maternal deaths (0.46%, 95% CI 0.18%–1.18%), two of which were due to hemorrhagic shock following CS in women with >3 previous CS, who appeared to have an abnormally invasive placenta (AIP). Maternal near misses were recorded in 32/868 (3.7%, 95% CI 2.6%–5.2%) of women, with a higher rate among those who had a previous scar (20/191, 10.5%, 95% CI 6.9%–15.6%) or those gave birth by CS (27/136, 19.9%, 95% CI 14.0%–27.3%). Uterine rupture occurred in 13/868 (1.5%, 95% CI 0.9%–2.5%) of women and AIP in 7/868 (0.8%, 95% CI 0.4%–1.7%) in the general population, while in women with a history of CS, uterine rupture occurred in 11/191 (5.8%, 95% CI 3.2%–10.0%) and AIP in 7/191 (3.7%, 95% CI 1.8%–7.4%), with blood transfusions required in all women.

**Table 3:** Maternal and perinatal outcomes of all births in Walikale

	Total *n*=868	Vaginal birth *n*=732 (%)	CS *n*=136 (%)	*P* value
**Maternal complications**				
**Maternal death**	4 (0.5)	1 (0.1)	3 (2.2)	<.01
**Maternal near miss** (composite, WHO-defined)	32 (3.7)	5 (0.7)	27 (19.9)	<.01
**Uterine rupture**	13 (1.5)	0 (0)	13 (9.6)	<.01
**Placental abruption**	6 (0.7)	0 (0)	6 (4.4)	<.01
**Abnormally invasive placenta**	7 (0.8)	1 (0.1)	6 (4.4)	<.01
**Severe hemorrhage**	52 (6.0)	28 (3.8)	24 (17.6)	<.01
**Hemorrhage requiring blood transfusion**	32 (3.7)	10 (1.4)	22 (16.2)	<.01
*One or two units of whole blood*	*20 (2.3)*	*8 (1.1)*	*12 (8.8)*	
*Three or more units of whole blood*	*12 (1.4)*	*2 (0.3)*	*10 (7.4)*	
**Maternal sepsis**	21 (2.4)	4 (0.5)	17 (12.5)	<.01
**Perioperative complications**				
Any severe peri- or postoperative complication		-	41 (30.1)	N/A
Peripartum hysterectomy		-	8 (5.9)	N/A
Relaparotomy		-	10 (7.4)	N/A
Bladder, intestinal injury, and/or fistula		-	4 (2.9)	N/A
Severe surgical site infection		-	10 (7.4)	N/A
Anaesthesia-related		-	1 (0.7)	N/A
**Perinatal complications**				
**Perinatal death**	53 (6.1)	32 (4.4)	21 (15.4)	<.01
Stillbirth	28 (3.2)	20 (2.8)	8 (5.9)	*.07*
Neonatal death	25 (2.9)	12 (1.6)	13 (9.6)	<.01
**Neonatal near miss** (composite, *n*=840) Asphyxia, neonatal infection, sepsis, intraventricular hemorrhage, respiratory distress (excluding stillbirths)	76 (9.0)	45 (6.3)	31 (24.2)	<.01
**Adverse neonatal outcome** (composite) Neonatal death, admission to neonatal department, preterm <37 wk, low birth weight <2500 grams	209 (24.1)	155 (21.2)	54 (39.7)	<.01

Severe hemorrhage requiring blood transfusion occurred in 22 (16.2%, 95% CI 10.9%–23.3%) women, of whom 18 (81.8%, 95% CI 61.5%–92.7%) had a history of CS. Relaparotomy occurred in 10 (7.4%, 95% CI 4.0%–13.0%) women and peripartum hysterectomy in 8 (5.9%, 95% CI 4.0%–13.0%); all women had a history of CS. The rate of severe surgical site infections was 7.4% (*n*=10/136, 95% CI 4.0%–13.0% with Robson Group 1 showing the highest incidence (26.3%, *n*=5/19, 95% CI 11.8%–48.8%). Perinatal mortality rate was 60 per 1000 total births (*n*=53/886 total births, 95% CI 46.0–77.4 per 1000 births), with a stillbirth rate of 31.6 per 1000 births (*n*=28/886, 95% CI 22.0–45.3 per 1000 births) and neonatal death rate of 29.0 per 1000 live births (*n*=25/861 live births, 95% CI 19.7–42.5 per 1000 live births). Perinatal mortality was higher among women with a previous scar (86 per 1000 births, *n*=17/197, 95% CI 54.6–133.8 per 1000 births) and by CS (145 per 1000 births, *n*=21/145, 95% CI 96.7–211.3 per 1000 births).

### Clinical case reviews

Table 4 summarizes the findings from clinical case review of all CS. In 10 of 17 CS (58.8%) performed in the second stage of labor, a vacuum-assisted birth was attempted. The decision-to-delivery interval was <60 minutes in 127 women (93.4%). The high proportion of CS performed under ketamine anaesthesia (58.5%, *n*=79) was due to shortages of spinal needles. The majority of CS were performed using midline incision (56.7%, *n*=77), as preferred by clinicians when women had a previous midline incision. Severe CS (hemorrhage, wound infections, relaparotomy) complications occurred in 30.1% (*n*=41/136, 95% CI 23.1%–38.3%).

**Table 4:** Clinical case review findings

	Total *n*=136 (%)
**Clinical factors**	
**Phase of labor**	
Not in labor	10 (7.4)
Latent phase	70 (51.5)
Active phase	56 (41.2)
First stage	39 (28.7)
Second stage	17 (12.5)
**Partograph completed**, *n*=56	51 (91.2)
**Membranes ruptured**	68 (50.4)
**Decision-to-delivery interval**	
<30 min	77 (56.6)
30–59 min	40 (36.8)
≥60 min	9 (6.6)
**Anaesthesia**	
Ketamine	79 (58.5)
Spinal	51 (37.5)
Spinal converted to ketamine	5 (0.6)
**Skin incision**	
Pfannenstiel	59 (43.3)
Midline/vertical	77 (56.7)
**Duration of CS**	
<45 min	31 (22.8)
45–59 min	56 (41.2)
60–89 min	36 (26.5)
≥90 min	13 (9.6)
**Clinical case review**	
**Indication**	
Absolute	68 (50.0)
Relative	57 (41.9)
No indication	11 (8.1)
**Quality of decision and timing to perform CS**	
At the right moment	90 (66.2)
Too soon	33 (24.3)
Too late	13 (9.6)
**Urgency level**	
Very urgent (strive for delivery <30 min)	44 (32.4)
Urgent (strive for delivery in 30–90 min)	65 (47.8)
Nonurgent	27 (19.9)
**Consent form completed**	123 (92.5)
**Documentation complete**	129 (96.3)

Clinical case reviews revealed that half of all CS (*n*=68) were performed for absolute indications, meaning a life-threating condition (*see* Additional File 5 for clinical case review outcomes per Robson group, assessing absolute or relative indication, “too soon” and “too late,” level of urgency). One in four CS (24.3%, *n*=33) were considered to have been performed “*too soon*” (without a good indication) while one in 11 (*n*=13, 9.6%) were deemed “*too late.*” The majority of CS classified as “too soon” (without a good indication) occurred in R1, R3, and R5.1, and R5.2. In these cases, labor was deemed nonprogressive; however, not all interventions were attempted to support labor progression before proceeding to CS. These interventions include ambulation, partograph use, bladder emptying, and oxytocin augmentation. Conversely, most CS classified as being conducted “too late” were in R5.3 (*n*=10/13), concerning women with history of >3 previous CS, who presented with uterine rupture (*n*=4) or in late stage of labor (*n*=6), leading to one maternal death and four stillbirths.

One in five CS (19.9%, *n*=27) were categorized as nonurgent. None of all CS conducted for >3 previous CS (*n*=34) were planned or conducted in a nonurgent setting.

Table 5 provides a summary of positive practices and recommendations (detailed version with all suggestions is found in Additional File 6). The strongest point was the rapid CS decision-to-delivery time, while key recommendations included increasing clinical supervision of CS decision-making and improving sensitization tubal ligations, especially for women with multiple previous CS.

**Table 5:** Positive practices and recommendations following CS clinical case reviews

	Positive practices		
	Patient and community factors	Referral system and access to care	Quality of clinical care
**Recorded in many cases** **in fewer cases**	Attended antenatal care Stayed at maternity waiting home until delivery due to high-risk pregnancy Arrival on time, in latent stage of labor, despite living >2 h from hospital Came with consent letter from husband to perform tubal ligation	Free hospital care for deliveries (MSF supported) Referral to hospital by health center before delivery in case of previous CS	Short interval between decision to perform CS and the birth Good surveillance of mother and baby, and protocols followed Availability of doctor around the clock Less experienced doctors and nurses ask a colleague for help Availability and good use of ultrasound enhanced decision-making and reduced severity of complications
	**Recommendations**		
	**Patient and community factors**	**Referral system and access to care**	**Quality of clinical care**
**Recorded in many cases** **in fewer cases**	Importance of knowing LMP/gestational age to reduce pregnancy risks Sensitization about risks of repeated CS and importance of contraception The harmful effects of traditional medication _(Kashisha)_ Allowance of birth companion would enhance cooperation and increase vaginal births Education about nutritional needs and anemia prevention during pregnancy	Make antenatal care free of charge and ensure availability of basic medications Put in place free ambulance (or motorcycle if not possible) for referral of emergency obstetric cases Implement referral criteria in health centee to aid decision-making for referral to maternity waiting home and hospital during pregnancy, labor, and postnatal period	Clinical supervision by experience obstetrician needs to be made possible for clinical decision-making and management of difficult cases Improving quality of ANC (staffing, availability of medication, vaccines, etc.) Management of prolonged labor and induction of labor needs improvement (with revision of local practice and introduction of new generation partograph) Quantify blood loss during childbirth and CS to prevent underestimation and implement local blood bank Improvement of fetal monitoring, especially in high-risk cases Enable option for referral of patient to higher center

A visual abstract and infographic summarizing the study’s findings in both English and French are provided in Additional File 7.

## Discussion

### Principal findings

A key finding of our study is that in Walikale, 22% of women who give birth at the hospital have a history of CS, and that the contribution of Robson group 5 to the overall CS rate (16%) is 50%. The VBAC rate is very high for a low-resource setting, with VBAC success in 71% for VBAC-1 50% for VBAC-2. Simultaneously, the complication rate of CS is also high, with many complications occurring in women with prior CS, such as uterine rupture (5.8%) and AIP (3.7%). These findings underscore the urgent need to prevent unnecessary first CS, ensure high-quality surgical care for complex CS, improve the safety of VBAC, ensure access to elective CS and tubal ligations, especially for women with history of >3 previous CS to reduce maternal and perinatal mortality.

### Results, clinical and research interpretation

The CS rate in Walikale hospital (16%) aligns closely with hospital-based rates reported across rural DRC, which range from 14% to 17% in rural areas, yet increase to 31% in urban Kinshasa.^20^^,^^21^^,^^29^, ^30^, ^31^ The hospital CS rate in our study is notably lower than in many other rural referral hospitals (eg, Malawi 20%, Tanzania 35%, South Africa 31%).^32^, ^33^, ^34^

However, hospital rates should be interpreted with caution, as they are influenced by the facility’s obstetric population. The 2015 WHO statement emphasizes that the optimal population-based CS rate is context-specific, though CS rates above 19% show no further correlation with improved maternal and perinatal outcomes.^35^, ^36^, ^37^, ^38^, ^39^ A recent publication by Zaigham et al^40^ highlights the immense global disparities in CS use and underscores the need for indication-based metrics (the “*why*”) next to the Robson classification to help global health experts and policymakers understand CS drivers and formulate targeted strategies to prevent both underuse and overuse.

The high prevalence of women with a previous scar in our study of 22% surpasses the average of 6% observed in hospitals in low-income countries, and though incomparable, even surpasses the 13% in high-income countries.^41^ The high number of women with a history of CS, and thus at high risk of complications in subsequent pregnancies, can be attributed to several factors. First, DRC has one of the highest fertility rates globally (one of four countries with an average of six or more children per woman in 2024), and thus many subsequent pregnancies after a previous CS, increasing the size of R5 over time.^42^ Second, with MSF providing free care for the past decades and more regional stability, there is increased access to healthcare and thus higher number of CS performed, especially in R1-4, which increased the size of R5 over time.^7^ Third, although exact numbers are unknown, there is a growing influence of the private sector, with CS performed for nonmedical reasons, incentivized by financial gain, a trend also observed in other settings.^43^^,^^44^ Lastly, contributing to these complexities, is the lack of clear guidelines for CS decision-making in low-resource settings. Increased access to internet and social media exposes healthcare providers to guidelines and practice originating from middle- and high-income settings, where CS is often performed for lower-threshold indications (eg, breech delivery, or eclampsia).^45^ These practices may be less suitable for low-income settings due to higher risks of complications and the long-term consequences associated with high fertility rates.^6^^,^^8^^,^^45^^,^^46^

In our study, 50% of all CS fell into Robson Group 5, a markedly higher proportion than reported in other low-resource settings (ranging from 7.7% to 34.5% in studies from Uganda, Burkina Faso, Ethiopia, and Nigeria—though most concern tertiary hospitals).^47^, ^48^, ^49^, ^50^, ^51^, ^52^ This high contribution is unlikely to be explained by historically elevated CS rates, as facility data show CS rates have remained below 15% since 2005, nor by low VBAC uptake, as VBAC rates in Walikale are already high. Instead, the most plausible explanation is the high fertility in this setting (average six children per woman).^7^ Once a woman has undergone a CS, she remains in Group 5 for all subsequent pregnancies, resulting in a large and growing population of women with a uterine scar and associated increased obstetric risk.

When looking at women with a uterine scar, the largest proportion, that is, 76.4% fall in Robson group 5, and an additional 24.6% (*n*=21/89) are found in Robson groups 6 to 10. The CS rate within Robson Group 5 in Walikale was relatively low (41.5%) compared to other facilities in low-, middle- and high-income countries, where rates exceed 63%.^41^ This reflects the practice in Walikale, that all women with one or two previous CS (with a fetus in cephalic presentation) generally attempt VBAC, with a high success rate (71% for VBAC-1, 50% for VBAC-2).^53^^,^^54^ A meta-analysis of VBAC-1 in sub-Saharan Africa showed an average VBAC rate of 34%, with a range from 14.2% to 84.7%.^55^ The high VBAC uptake in Walikale is likely influenced by cultural preferences favoring vaginal birth. Additionally, MSF protocols promote VBAC in conflict settings, especially when fertility rates are high, as future deliveries may not occur in a facility, and thus supporting VBAC when clinically “safe” is preferred to reduce the long-term risks associated with repeated CS. The reason for the previous CS (eg, uterine rupture) and the type of uterine incision (eg, classical) are often unknown, limiting the ability to assess VBAC safety on an individual basis. Moreover, women are generally not counselled on choosing between elective repeat CS and VBAC, and therefore may not fully understand the associated risks of VBAC and/or the cumulative risks of multiple CS. This highlights a substantial opportunity to improve counselling and decision-making support.

While providing VBAC in suboptimal conditions in low-resource settings may be considered less safe than an elective CS, and could be an ethical concern, the benefits of avoiding multiple CS scars in countries with high fertility rates outweighs these risks, as Kalisa et al demonstrated in a rural referral hospital in Rwanda.^56^^,^^57^ The complexity of offering VBAC in a conflict-affected, low-resource setting is illustrated by our own findings, where two maternal deaths occurred among women with multiple previous CS, both resulting from massive hemorrhage due to AIP. While VBAC can reduce the cumulative risks associated with repeated CS, its safety depends on timely recognition of complications and the ability to intervene rapidly (conditions that are difficult to guarantee in settings without continuous fetal monitoring, blood availability, or adequate surgical capacity). Although ethical concerns arise when promoting VBAC where intrapartum surveillance is limited, the countervailing principle of “do no further harm” highlights the need to avoid exposing women to the escalating long-term risks of multiple CS. Given the limited evidence available to guide decision-making in low-resource settings, clinicians must carefully weigh the short-term risks of offering VBAC against the longer-term harms of planned repeat CS.^55^

The priorities for the hospital of Walikale should therefore not be to increase VBAC rates but rather prevent the first unnecessary CS (CS performed “too soon”/without a good indication) and enhance VBAC safety (improved monitoring, more careful VBAC case selection). This can be achieved by implementing clear guidelines on CS indications tailored to low-resource contexts, decision-making by at least two clinicians, planning necessary elective CS during ANC, implementing CS case reviews, improving maternal and fetal monitoring, and ensuring 24/7 access to high-quality surgical care and blood products.^4^^,^^8^^,^^57^

The high rates of maternal and perinatal complications observed in our study closely align with findings from similar low-resource setting.^9^^,^^48^^,^^58^^,^^59^ Similar to our figures, a large multi-country study in sub-Saharan Africa reported very high rates of maternal death (0.5%) and severe complications after CS (17.4%), more than 50-fold higher than in high-resource settings.^55^^,^^58^ As mentioned earlier, the complications in our study mostly involved uterine ruptures, AIP and severe hemorrhage requiring blood transfusions. These were largely attributable to sequelae of a history of multiple CS (often 3, 4, 5, or even 6 CS). In Walikale, the risk of uterine rupture (1.5% in the general population and 5.8% among women with a prior CS) far exceeds the highest prevalence reported in another WHO multi-country study (2.5% among women with prior CS).^60^ Plausibly explained by the double burden: delayed presentation with obstructed labor (5/13 ruptures, including two women without previous CS and three women with history of >3 previous CS) and high-risk VBAC without fetal monitoring (8/13 ruptures). However, increasing primary CS rates is unlikely to be a solution, given the substantial surgical risks in low-resource settings, the future risk of AIP observed in this study, and the likelihood that some women will deliver without skilled attendance in subsequent pregnancies. Key priorities include preventing the first unnecessary CS, ensuring blood availability, establishing timely referral systems, improving VBAC safety through adequate staffing and monitoring, and strengthening surgical capacity, skills, and supervision.

There is also a high prevalence of prenatal morbidities (eg, malnutrition, anemia, malaria, HIV, fetal growth restriction) and limited availability of resources to ensure adequate monitoring and management of complications.^9^^,^^41^^,^^60^ Despite the availability of free care and high ANC attendance in Walikale, women with high-risk pregnancies are currently poorly identified. For example, women with a history >3 CS were never planned for elective CS, only a handful received a third-trimester ultrasound for placenta localization, and few received tubal ligation. This study highlights the crucial need to improve the quality of ANC through staff capacity building and strengthening of the referral system, alongside ensuring the provision of essential medications and vaccinations.^17^^,^^27^^,^^61^^,^^62^ Additionally, community sensitization efforts can encourage women with risk factors to seek care in a timely manner and to consider contraceptive options such as tubal ligation, especially after multiple previous CS. The low uptake of contraception in Walikale is consistent with patterns observed elsewhere in the DRC and reflects a substantial unmet need.^63^ Key barriers include limited awareness, misconceptions, male partner disapproval, and insufficient provider expertise, while cultural norms and high child mortality also contribute. These findings align with evidence from North Kivu and multi-country studies across sub-Saharan Africa.^63^^,^^64^ Other possible contributing factors to the high complication rate include the quality of prior CS, short inter-pregnancy intervals, and onset of labor at home. Lack of quality of care in health centers, untimely referral, and inadequate monitoring may have impacted the outcomes, but were not assessable in our study.^27^^,^^49^^,^^50^^,^^61^^,^^62^

Although there is no international consensus on assessing the appropriateness of CS decisions and indications, this study’s finding that one in four CS were performed “*too soon*” (and were thus potentially unnecessary) aligns with similar studies conducted in facilities in Malawi (26% with “unsupported indications”) and Namibia (20% with “indications not in accordance”).^65^^,^^66^ While absolute CS indications, such as a placenta previa or uterine rupture, are clear-cut, many CS indications, especially for fetal indications, require clinical judgment based on limited evidence, or evidence derived from high-income countries and not feasible to implement low-income contexts.^46^^,^^65^ Clinicians in these settings would greatly benefit from guidelines on CS indications tailored to their specific setting to support decision-making taking these contextual factors into account.^45^^,^^46^ Such guidelines could, for example in Walikale, recommend abstaining from CS for fetal indications when the estimated weight is below 1500 grams until neonatal care facilities and survival rates improve.

Lastly, although the Robson classification is a globally endorsed tool and helpful in prioritizing interventions to improve quality of care in low-resource settings, its implementation is challenging in these settings as most women do not know their gestational age.^67^ Many women present late in pregnancy, and those who attend early ANC are seen at primary health centers where assessments rely solely on abdominal palpation, without access to ultrasound. As a result, classification into Robson groups is based on rough clinical estimates (eg, newborn appearance and birth weight), impairing reliability of Robson 10. This reflects a broader challenge in such settings rather than a limitation unique to our study, highlighting the need to adapt Robson implementation for low-resource contexts.

### Strengths and limitations

Our study is unique in that it was conducted in a conflict-affected humanitarian setting, a context in which the Robson classification has rarely been applied. Although the classification itself is not novel, its use in this setting provides important new insights. Moreover, our study goes beyond standard Robson reporting by examining complication prevalence and conducting a quality-of-care assessment for every CS, providing a comprehensive overview of CS practice, with critical insights and actionable recommendations for policymakers and clinicians in similar settings.

Nonetheless, several limitations must be acknowledged. First, despite our robust data collection and cross-checking, the retrospective nature of the study may have introduced information bias. Second, several variables were unavailable or missing from the medical record, such as maternal nutrition status, socioeconomic status, number of ANC visits, potentially limiting the completeness of our analyses. Gestational age, which is crucial for distinguishing Robson group 10 from other groups, was fully reported but required estimation using clinical parameters in approximately half of the cases. This approach, while practical in low-resource settings, is less accurate than first-trimester ultrasound dating and may have affected group classification (underestimating group 10). Third, to ensure data quality and enable detailed clinical case reviews, the study could only be conducted in a single center and over a limited period due to resource constraints. This resulted in a relatively modest sample size, and although adequate for our research questions, it was insufficient to conduct multivariate regression analyses and assess risk factors for adverse outcomes, either overall or within key subgroups such as Robson categories or women with previous CS. Such analyses would be valuable for identifying drivers of complications and should be prioritized in future, larger-scale studies. Fourth, the clinical case review included subjective variables, as there is no available “gold standard” (such as the assessment of urgency and CS clinical indications). They were conducted by clinicians working at the study facility, with the risk of biases (based on their experience). Yet, the qualitative aspect provides a more nuanced and comprehensive understanding of the clinical realities behind the numerical data, and local clinicians are best suited to develop feasible, context-specific recommendations.

## Conclusion

The CS rate in the hospital of Walikale is 16%. A large proportion, that is, 22%, of women giving birth in this setting have a history of CS. The majority of CS are performed in women with a history of CS. At the same time, there is a high VBAC rate of 71% (VBAC-1) and 50% (VBAC-2), much higher than in other low-resource settings. The rate of maternal and perinatal complications is high, especially in women with a history of 3 or more CS. Improving the quality of ANC by planning elective CS and discussing tubal ligation is crucial to improve outcomes.

Future studies should specifically evaluate risk factors and outcomes in women with prior CS, given their substantial contribution to morbidity and mortality. Finally, settings such as Walikale urgently require clear, context-specific guidelines on CS indications and CS case reviews. Application of the Robson classification alone is insufficient for assessing the clinical appropriateness of CS, and expanding the tool with the addition of quality indicators, as attempted in this study, is essential to support clinicians and policymakers in optimizing CS use and ultimately reducing maternal and perinatal mortality.

## Consent for publication

No consent for publication was necessary.

## CRediT authorship contribution statement

**Geoges Lubuto Bushu:** Writing – review & editing, Writing – original draft, Methodology, Formal analysis, Conceptualization. **Christophe Kambale:** Writing – review & editing, Methodology, Formal analysis, Conceptualization. **Seraphin Kikwabantu:** Writing – review & editing, Methodology. **Faida Boelongo Benedite:** Writing – review & editing, Methodology. **Wolfgang Weber:** Writing – review & editing, Supervision, Methodology, Formal analysis. **Benjamin O. Black:** Writing – review & editing, Validation, Supervision, Conceptualization. **Kim J.C. Verschueren:** Writing – review & editing, Writing – original draft, Visualization, Validation, Supervision, Methodology, Investigation, Data curation, Conceptualization.

## Appendix Supplementary materials

Image, application 1

Image, application 2

Image, application 3

Image, application 4

Image, application 5

Image, application 6

Image, application 7

## Footnotes

Supplementary material associated with this article can be found in the online version at doi:10.1016/j.xagr.2025.100586.
