# Supplementary material for: Caesarean section Robson classification, complications, and lessons learned in a rural hospital in Walikale, North Kivu, Democratic Republic of Congo: a cross-sectional study
Source: AJOG Glob Rep. 2025 Nov 23;6(1):100586. doi: 10.1016/j.xagr.2025.100586 (PMC12771099; doi:10.1016/j.xagr.2025.100586)
Supplement: Supplementary file 1 [file mmc1.docx]

**Additional file 1**. Robson classification system ^1-5^


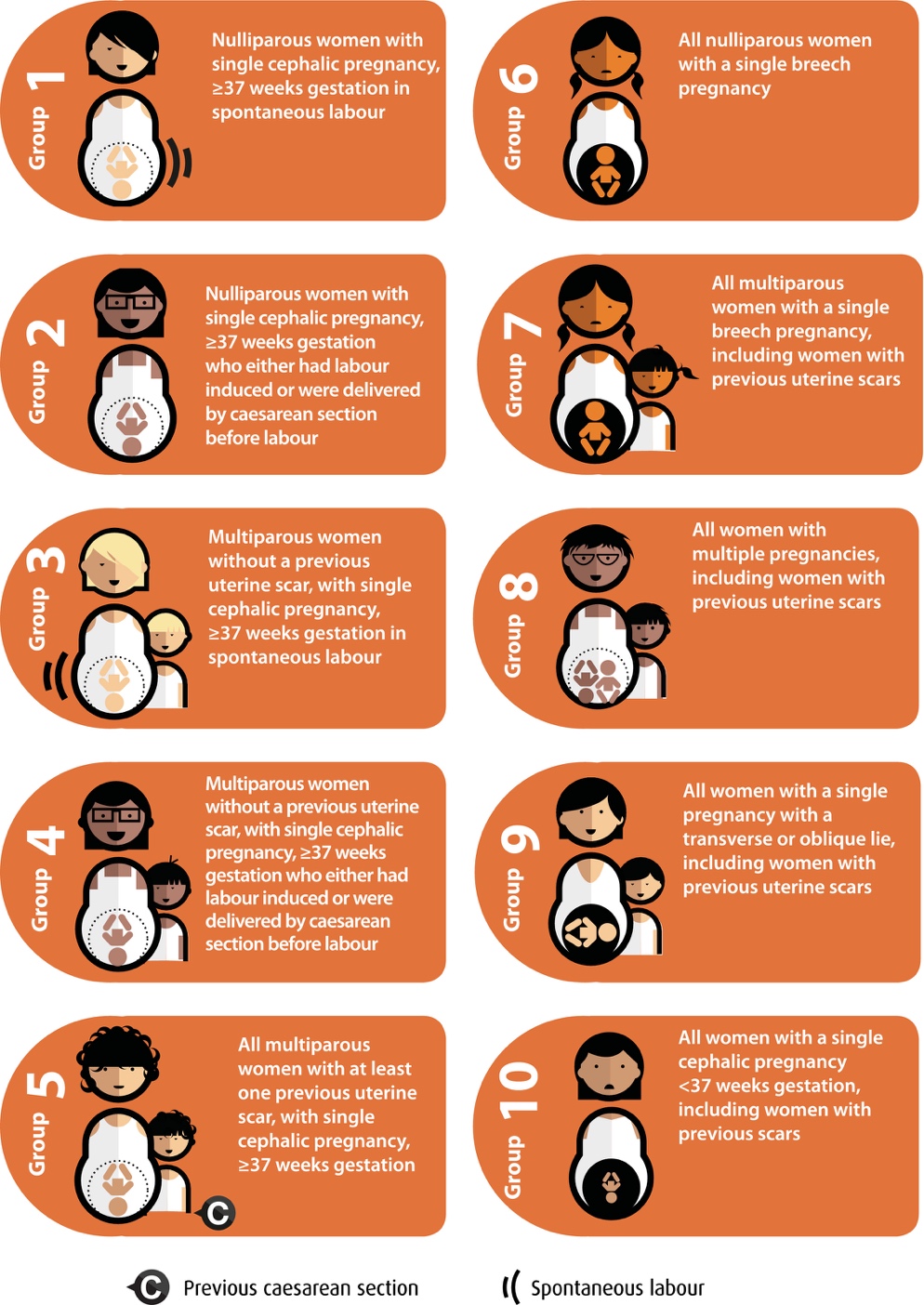


Unknown gestation:

>2500g = term, <2000g = preterm

2000–2499g = clinical assessment

(see additional file 2 for elaboration)

References

1. **Betrán AP, Ye J, Moller AB, Zhang J, Gülmezoglu AM, Torloni MR.** The Robson classification system for caesarean sections: A systematic review of the literature. PLOS ONE, 2016: 11(5), e0158180. DOI: 10.1371/journal.pone.0158180.
2. Opiyo, N., Torloni, M., & Robson, M. WHO's Robson platform for data-sharing on caesarean section rates. Bulletin of the World Health Organization, 2022: 100(5), 352-354. DOI: 10.2471/BLT.21.287742.
3. **Robson, MS.** Classifying rates of caesarean section by obstetric outcome. Journal of Obstetrics and Gynaecology, 2001: 21(3), 282-290. DOI: 10.1080/01443610120068157.
4. Robson, M. Use of indications to identify appropriate caesarean section rates. Lancet Global Health, 2018: 6(8), e820-e821. DOI: 10.1016/S2214-109X(18)30319-X.
5. Robson Classification Platform. Accessed October 15, 2024. <https://robson-classification-platform.srhr.org/>
6. **World Health Organization.** Robson classification: Implementation manual. Geneva: WHO. Accessed on October 15, 2024. <https://www.who.int/publications/i/item/9789241513198>.
