# Supplementary material for: Caesarean section Robson classification, complications, and lessons learned in a rural hospital in Walikale, North Kivu, Democratic Republic of Congo: a cross-sectional study
Source: AJOG Glob Rep. 2025 Nov 23;6(1):100586. doi: 10.1016/j.xagr.2025.100586 (PMC12771099; doi:10.1016/j.xagr.2025.100586)
Supplement: Supplementary file 2 [file mmc2.docx]

**Additional file 2. Variables, definitions and categorization**

**Variables collected for all births** (derived from medical records)

**Maternal characteristics:**

- Maternal age, categorised into adolescence (<20 years), adulthood (20 - 34 years), and advanced maternal age 35 years and older).^1^
- Village name and axe, and distance to hospital in minutes by motorcycle (<30 minutes, 30-59 minutes, 60-119 minutes, 120 minutes and more).^2^
- Parity, categorised into primiparity - women with no previous births (P0), low multiparity - women with one to four previous births (P1-4), grand multiparity – women with five to nine previous births (P5-9) and great grand multiparity – women with 10 or more previous births (>P10).^3^
- Previous CS (yes/no), number of previous CS, whether there was a vaginal birth after the CS, and the interval between the previous CS and the current pregnancy.^4^
- ANC visits (yes/no) and number of visits
- Blood group (A/B/AB/O) and rhesus (positive/negative)
- HIV status (positive/negative)
- Use of traditional medicines (yes/no)^5^
- Singleton or multiple (twins, triplets)
- Foetal presentation (cephalic, breech, or transverse)
- Admission to the maternity waiting home before delivery (yes/no)^6^

**Childbirth information:**

- A birth is defined as the complete expulsion or extraction of a foetus, regardless of signs of life, at >**22 weeks of gestation** or weighing > **500 grams.** While foetuses between 22-28 weeks and between 500-1000 grams are generally not viable in Walikale and other low resource settings, the WHO, ICD-10 and ICD-11 recommend this standardized definition to ensure international comparability.^7,8^Date of birth
- Time of birth
- Mode of delivery (vaginal, instrumental, CS)
- Gestational age, categorised as very preterm (less than 32 weeks), moderate preterm (32 weeks to 33 weeks and 6 days), late preterm (34 weeks to 36 weeks and 6 days), term (37 weeks to 41 weeks and 6 days), and post-term (42 weeks and beyond).^9^ The gestational age was based on the last menstrual period (LMP), as no woman had an ultrasound before 28 weeks of gestation. If the gestational age was unknown, the variable was recorded as missing. For Robson classification and the definition of adverse perinatal outcomes, birth weight and clinical assessment were used to classify infants as preterm or term.
- Babies weighing more than 2500 grams were considered term, while those under 2000 grams were classified as preterm. Infants weighing between 2000 and 2499 grams were clinically assessed by the to determine whether they were term but dysmature or preterm, with this distinction recorded in the medical file. Preterm babies had a head and abdominal circumference appropriate for their gestational age, thin skin with fused eyelids, short and soft nails, lanugo, and poor muscle tone with extended limbs. In contrast, dysmature term babies exhibited a malnourished appearance, with a head circumference typical of a term infant but a reduced abdominal circumference, often with visible ribs. They also had loose, dry skin—often meconium-stained—along with long hair, long nails, and a thin umbilical cord.^9-12^Labour induced (yes/no)^11^
- Oxytocin augmentation (yes/no)^11^
- Episiotomy performed (yes/no)^11^
- Active management of third stage of labour (yes/no)^11^
- Contraception provided postpartum (yes/no) and tubal ligation (yes/no)

**Perinatal characteristics**

- Sex (male or female)
- Birth weight, categorised as very low birth weight (<1500 grams), low birth weight (1500-2499 grams), and normal birth weight (2500 grams or more).^12^Apgar score at 1, 5 and 10 minutes based on five criteria of each 0-2 points. Asphyxia is defined as a 1-minute Apgar <5 and/or 5-minute Apgar score <7.^13^
- Admission to neonatal unit (yes or no) and number of days

**Maternal complications:**

- Maternal death^14^ or near miss^15,16^
  - Maternal deathis a woman who died during pregnancy or within 42 days after delivery. The maternal mortality ratio was not reported, as the study population was small (thus, large confidence interval) to provide a reliable estimate.
  - Maternal near miss refers to a woman who nearly dies but survives a life-threatening complication during pregnancy, childbirth, or within 42 days of the end of pregnancy. The WHO near miss tool criteria are based on specific markers of **severe organ dysfunction.** We used an adapted version of the WHO tool, designed specifically for Sub-Saharan African following a Delphi study conducted in 2017.^16^ The amendments improve detection of maternal near misses in low-resource settings and include the following clinical criteria: eclampsia, sepsis, uterine rupture, and blood transfusion of two or more units (instead of five or more units).
- Uterine rupture is the complete tear of the uterine wall.^17^
- Eclampsia is defined as one or multiple generalised convulsions in a woman with preeclampsia, characterised by hypertension and proteinuria, typically after 20 weeks of gestation and up to 10 days postpartum.^17^
- Placental abruption is the premature separation of the placenta from the uterine wall before birth of the foetus, leading to significant maternal and perinatal morbidity, and can be either partial (only a proportion, foetus can be alive) or complete (foetus is generally dead).
- Abnormally invasive placenta (AIP), or placenta accreta spectrum (PAS), refer to disorders where the placenta invades into the myometrium, with placenta accreta does not penetrate it, placenta increta invades and penetrates and placenta percreta invades surrounding organs, such as the bladder.^18^
- Severe haemorrhage concerns blood loss greater than 1000 mL during or after childbirth. As blood loss is not quantified in our setting, an estimation of 500 mL with clinical signs of shock or the need for blood transfusion was also considered severe haemorrhage.^19^
- Blood transfusion (yes/no) and the number of whole blood units transfused.
- Maternal sepsis is characterised by the presence of infection alongside signs of systemic inflammation, includes fever, tachycardia, tachypnoea and altered mental status. The site of infection is recorded as: uterus (endometritis), urinary tract, surgical site (after CS) or other (pneumonia, opportunistic infections cause by HIV). Additionally, all cases of intrapartum and immediate postpartum symptomatic malaria are reported.^20^

**Perinatal complications:**

- Perinatal death^21^ refers to a stillbirth^22^ or neonatal death^23^
  - Stillbirth (≥ 28 weeks or ≥ 1,000 grams) refers to an infant born without signs of life. Stillbirths can occur either antepartum (before labour, characterised most often as ‘macerated stillborn’) or intrapartum (during labour, characterised as ‘fresh stillborn’).^22^
  - Neonatal death refers to the death of a liveborn infant within the first 28 days of life. Early neonatal deaths occur during the first 7 days, late neonatal deaths occur day 8-27.^22-24^
- Neonatal near miss includes asphyxia, sepsis, intraventricular haemorrhage or respiratory distress requiring admission to neonatal care unit. In contrast to ‘adverse neonatal outcomes’ low birth weight and preterm birth are not included in this definition.^9-13,22,-24^
- Adverse neonatal outcomesrefer to negative events or conditions that affect newborns during the first 28 days of life, defined as either neonatal death, admission to neonatal care unit, preterm birth (before 37 weeks of gestation), and/or a low birth weight (<2500 grams).^9-13,22-24^

**Indicators** (derived from abovementioned collected variables)

- Robson 10 Groups classifies each birth using parity (primiparous/multiparous), foetal number (singleton/multiple), foetal presentation (cephalic/breech/transverse), previous CS, and gestational age (term/preterm), see additional file 1^25,26^
- The VBAC success rate indicated the proportion of women who attempt a vaginal birth after a previous CS and successfully deliver without requiring an emergency CS. **VBAC-1** describesa vaginal birth after one previous caesarean, while **VBAC-2** refers to a vaginal birth after two previous caesareans.
- The maternal death ratio is calculated as the number of maternal deaths per 100,000 live births.^14^ The maternal near miss rate as the number of maternal near miss per 1000 live births.^15^ The perinatal death rate is calculated as the number of perinatal deaths per 1000 births.^21,22^ The stillbirth rate as the number of antenatal and intrapartum stillbirths per 1000 births.^21,22^ The neonatal death rate as the number of neonatal deaths per 1000 live births.^21^

**Variables collected for all CS** (derived from medical records)

**Clinical variables**

- Leading primary CS indication as recorded by the treating physician^26-28^– this is the primary medical reason and most significant factor that led to the decision to perform the CS. For example, a woman with one or two previous CS and suspected foetal distress - the primary indication is foetal distress, as otherwise the clinician would have allowed vaginal delivery.
- Type of skin incision (vertical midline, Pfannenstiel)
- Type of anaesthesia (ketamine, spinal anaesthesia, conversion from spinal to ketamine)
- Membranes (ruptured or intact)
- Dilation in cm (0 to 10 cm)
- Phase of labour (no labour, latent phase, active phase)^29^
- Stage of active labour: first stage (5 to 9 cm dilation) or second stage (10 cm dilation)^29^

**Documentation variables**

- Interval between decision and delivery (time in minutes)^30,31^
- Duration of procedure (time in minutes)^32^
- Partograph (complete, incomplete, not present, not applicable (latent phase))^29^
- Consent (complete, partially completed, not present)^33^
- Documentation (complete, incomplete)

**Perioperative and postoperative complications related to CS**

- Severe peripartum haemorrhage during surgery with blood loss greater than 1000 mL and/or clinical signs of shock and/or the need for blood transfusion.^19^
- Peripartum hysterectomy is the surgical removal of the uterus, which is a major surgical intervention, typically performed for uncontrolled bleeding without any other options remaining, uterine ruptures that are not repairable, abnormal placentation and severe uterine infections.^20^
- Anaesthesia-related complications refer to adverse effects resulting from the administration of anaesthesia (ketamine and/or spinal) during the CS, which include respiratory depression or cardiovascular instability in a setting with limited monitoring and without the ability to provide general anaesthesia.
- Trauma to other organ (bladder or bowel injury), relaparotomy and obstetric fistula
- Surgical site infection, either mild and remaining to the skin and subcutaneous tissue, or severe, with extension into fascia, muscle, and peritoneum.^34^

**Clinical case review variables (n=136 cases)** (derived from case review by 5 of the authors)

- **Indication of CS**, based on the 2019 MSF obstetric guidelines **^2835^**
  - *Absolute indications* for CS directly threaten the life of the mother ^35^
    - A history of three or more caesareans
    - Uterine rupture
    - Severe antepartum haemorrhage (tachycardia, hypotension) not controlled
    - Total placenta previa
    - Fixed malpresentation (shoulder, forehead, face with posterior chin)
    - Absolute foetopelvic disproportion (partograph showing failure to progress in the active phase of labour despite good uterine dynamics) and inability to perform instrumental extraction

Absolute foetopelvic disproportion is distinct from “prolonged labour”, which is defined as labour crossing the action line on the partograph without all available interventions (such as rupture of membranes, oxytocin augmentation and/or instrumental delivery) being attempted. In these cases, it is classified as a relative indication or no indication for CS.

- - *Relative indications* involve situations where vaginal delivery may have been possible but was not attempted due to potential risks to the baby.^35^ This category also includes CS performed for prolonged labour in which not all interventions were attempted, and foetal distress assessed by doppler (persistent foetal heart rate >160/min and <100/min or late decelerations). Other relative indications include failed induction in which not all interventions were attempted, cord prolapse, breech, twin with longitudinal presentation of first twin, growth restriction, placental abruption with stable maternal condition, maternal fever and suspected chorioamnionitis without signs of maternal sepsis.

In low-resource settings with limited access to care, high fertility rates and significant risks of maternal complication of CS (short and long term), these factors are weighed against the potential benefit of the procedure for the baby.

- - *No indications for CS* refer to cases where there was no threat to either the mother or the child, and a (longer) trial of labour should have been considered before opting for a CS.
- **Quality of decision and timing to perform CS ^36-39^** was
  - *At the right moment:* The decision to perform the CS was timely, with no other feasible or useful interventions that could be attempted without endangering the life of the mother and/or baby.
  - *Too soon:* The CS may have been avoidable with more patience or other interventions, as the lives of the mother and baby were not at risk, they can suggest CS ‘overuse’ or even be considered unjustified; or
  - *Too late:* The decision to perform the CS was delayed, putting the life of the mother and/or baby at risk by not acting sooner.^36-39^
- **CS urgency level** refers to the level of urgency with which the CS should be performed, as determined during the clinical case review. This evaluation is based on the indication and clinical findings, without consideration of the actual decision-to-delivery timing.^40,41^
  - *Very urgent:* CS should be performed within 30 minutes of decision, as there is acute maternal or foetal compromise (e.g. suspected uterine rupture, placental abruption with maternal/foetal distress, three prior CS and now in active stage of labour, failed instrumental delivery, acute foetal distress such as persistent foetal bradycardia, or cord prolapse); or
  - *Urgent*: CS should be performed within 30 to 90 minutes. While there is no immediate maternal or foetal compromise, further delay could pose risks (e.g. three prior CS and now in latent stage of labour, foetal malpresentation, absolute foetopelvic disproportion, suspected chorioamnionitis); or
  - *Non-urgent*: Immediate delivery is not necessary, as there are no risks to the mother or baby associated with delaying the CS beyond 90 minutes. Elective CS, which represent only a small proportion in low-income settings, are also classified as non-urgent. ^40,41^
- **Positive practices to be maintained for high-quality care** is an open variable and includes observations following the clinical case review. **Recommendations for improving care in similar cases** are open variables, with a maximum of three recommendations per case.^36-39,42,43^
  - One to three points are mentioned per element, and they consist of the most remarkable points according to the expert study team. Both the positive practices and recommendations use the Three-Delays-Model^44^ as a framework to categorise them: patient / community factors (first delay), accessibility of care and referral system (second delay) and qualitative and appropriate care in hospital (third delay). As data was based on clinical records and not on interviews or information from health centres, the data and information obtained regarding the first and second delays were limited.

References

1. World Health Organization. Adolescent pregnancy. Fact sheet, January 2020. Accessed October 15, 2024. <https://iris.who.int/bitstream/handle/10665/112320/?sequence=1>
2. World Health Organization. Service Availability and Readiness Assessment (SARA): An annual monitoring system for service delivery. Geneva: WHO; 2015. Accessed October 15, 2024. <https://www.who.int/data/data-collection-tools/service-availability-and-readiness-assessment-(sara)>
3. Cunningham FG, Leveno KJ, Bloom SL, et al. Williams Obstetrics, 25th edition. McGraw Hill, 2018.
4. American College of Obstetricians and Gynecologists. ACOG Practice Bulletin No. 205: Vaginal Birth After Cesarean Delivery. *Obstetrics & Gynecology*. 2019;133(2).
5. Shewamene Z, Dune T, Smith CA. The use of traditional medicine in maternity care among African women in Africa and the diaspora: a systematic review. *BMC Complement Altern Med*. 2017 Aug 2;17(1):382. doi: 10.1186/s12906-017-1886-x.
6. Medecins Sans Frontieres. Walikale, North Kivu: a “Host Village” to prevent maternal and infant mortality. Published on April 4, 2024. Accessed October 15, 2024. <https://msf.or.ke/news-and-resources/news/walikale-north-kivu-host-village-prevent-maternal-and-infant-mortality>.
7. Blencowe H, Hug L, Moller AB, You D, Moran AC. Definitions, terminology and standards for reporting of births and deaths in the perinatal period: International Classification of Diseases (ICD-11). *Int J Gynaecol Obstet.* 2025 Jan;168(1):1-9. doi: 10.1002/ijgo.15794.
8. **World Health Organization.** (2016). WHO recommendations on antenatal care for a positive pregnancy experience. World Health Organization. [https://apps.who.int/iris/handle/10665/250796](https://apps.who.int/iris/handle/10665/250796)
9. Jason O. Gardosi. Prematurity and fetal growth restriction. *Early Human Development.* 2005;81(1):43-49. https://doi.org/10.1016/j.earlhumdev.2004.10.015.
10. World Health Organization. Managing Complications in Pregnancy and Childbirth: A Guide for Midwives and Doctors. World Health Organization, 2007. Accessed October 15, 2024. <https://www.who.int/publications/i/item/9789241595681>.
11. World Health Organization. (2012). The WHO application of ICD-10 to deaths during pregnancy, childbirth, and puerperium: ICD MM. Geneva. ISBN: 978 92 4 154845 8. Accessed October 15, 2024. <https://iris.who.int/bitstream/handle/10665/70929/9789241548458_eng.pdf>
12. World Health Organization. (2011). Evaluating the quality of care for severe pregnancy complications: The WHO near-miss approach for maternal health. Geneva. ISBN 978 92 4 150222. Accessed October 15, 2024. <https://iris.who.int/bitstream/handle/10665/44692/9789241502221_eng.pdf;jsessionid=B2EA8E2B146622479D6D559BCBFDAAD3?sequence=1>
13. Tura A, Stekelenburg J, Scherjon S. Adaptation of the WHO maternal near miss tool for use in sub–Saharan Africa: An international Delphi study. BMC Preg and Childb. 2017: 17, 445. doi:10.1186/s12884-017-1640-x.
14. Schaap T, Bloemenkamp K, Deneux-Tharaux C, Knight M, Langhoff-Roos J, Sullivan E, van den Akker T; INOSS. Defining definitions: a Delphi study to develop a core outcome set for conditions of severe maternal morbidity. *BJOG.* 2019 Feb;126(3):394-401. doi: 10.1111/1471-0528.14833.
15. American College of Obstetricians and Gynecologists (ACOG). Practice Bulletin No. 183: Postpartum Hemorrhage. *Obstetrics & Gynecology*. 2020: 135(2), e168-e186. DOI: 10.1097/AOG.0000000000003601.
16. World Health Organization. Maternal sepsis: A WHO systematic review of the literature, 2015. Accessed October 15, 2024. <https://www.who.int/publications/i/item/maternal-sepsis>.
17. World Health Organization. The WHO application of ICD-10 to deaths during the perinatal period: ICD-PM, 2016. Accessed October 15, 2024. <https://www.who.int/publications/i/item/9789241549752>
18. World Health Organization. Making every baby count: Audit and review of stillbirths and neonatal deaths, 2016. Geneva: WHO. Accessed October 15, 2024. <https://www.who.int/publications/i/item/9789241511224>.
19. World Health Organization. Neonatal and perinatal mortality: Country, regional and global estimates, 2006. Geneva: WHO. Accessed October 15, 2024. <https://www.who.int/publications/i/item/9789241563209>.
20. World Health Organization. Standards for improving the quality of care for small and sick newborns in health facilities, 2022. Geneva: WHO. Accessed October 15, 2024. <https://www.who.int/publications/i/item/9789240068774>.
21. Robson MS. **Classifying rates of cesarean section by obstetric outcome.** Journal of Obstetrics and Gynaecology. 2001: 21(3), 282-290. DOI: 10.1080/01443610120068157.
22. World Health Organization. Robson classification: Implementation manual, 2017. Geneva: WHO. Accessed October 15, 2024. <https://www.who.int/publications/i/item/9789241513198>.
23. World Health Organization. WHO Statement on Caesarean Section Rates, 2015. Geneva: WHO. Accessed October 15, 2024. <https://www.who.int/reproductivehealth/publications/maternal_perinatal_health/cs-statement/en/>.
24. Groen R, Trelles M, Caluwaerts S, et al. A cross-sectional study of indications for cesarean deliveries in Médecins Sans Frontières facilities across 17 countries. International Journal of Gynaecology and Obstetrics, 2015: 129(3), 231-235. DOI: 10.1016/j.ijgo.2014.12.008.
25. World Health Organization. Partograph in management of labor: WHO recommendations, 2019. Geneva: WHO. Accessed October 15, 2024. <https://www.who.int/publications/i/item/9789241516182>.
26. May RL, Clayton MA, Richardson AL, Kinsella SM, Khalil A, Lucas DN. Defining the decision-to-delivery interval at caesarean section: narrative literature review and proposal for standardisation. *Anaesthesia.* 2022 Jan;77(1):96-104. doi: 10.1111/anae.15570.
27. Soltanifar S, Russell R. The National Institute for Health and Clinical Excellence (NICE) guidelines for caesarean section, 2011 update: implications for the anaesthetist. Int *J Obstet Anesth.* 2012;21(3):264–72. doi: 10.1016/j.ijoa.2012.03.004
28. Soergel P, Jensen T, Makowski L, von Kaisenberg C, Hillemanns P. Characterisation of the learning curve of caesarean section. *Arch Gynecol Obstet*. 2012 Jul;286(1):29-33. doi: 10.1007/s00404-012-2230-9.
29. Bakker W, Zethof S, Nansongole F, Kilowe K, van Roosmalen J, van den Akker T. Health workers' perspectives on informed consent for caesarean section in Southern Malawi. *BMC Med Ethics*. 2021 Mar 29;22(1):33. doi: 10.1186/s12910-021-00584-9.
30. World Health Organization. Global guidelines for the prevention of surgical site infection, 2016. Geneva: WHO. Accessed October 15, 2024. <https://www.who.int/publications/i/item/9789241549886>.
31. Médecins Sans Frontières. Guide: Essential obstetric and newborn care, 2019. Accessed October 15, 2024. <https://medicalguidelines.msf.org/en/viewport/ONC/english/6-4-caesarean-section-51417253.html>
32. Miller S, Abalos E, Chamillard M. Beyond too little, too late and too much, too soon: A pathway towards evidence-based, respectful maternity care worldwide. The Lancet. 2016: 388(10056), 2176-2192. DOI: 10.1016/S0140-6736(16)31472-6.
33. Dekker L, Houtzager T, Kilume O, Horogo J, van Roosmalen J, Nyamtema AS. Caesarean section audit to improve quality of care in a rural referral hospital in Tanzania. *BMC Pregnancy Childbirth.* 2018 May 15;18(1):164. doi: 10.1186/s12884-018-1814-1.
34. Heemelaar S, Nelissen E, Mdoe, P. Criteria-based audit of caesarean section in a referral hospital in rural Tanzania. Tropical Medicine & International Health. 2016: 21(4), 525-534. doi:10.1111/tmi.12683.
35. **Gordon A, Stokes C.** Assessing the quality of decision-making in cesarean section: A clinical audit. Journal of Obstetrics and Gynaecology, 2017: 37(2), 220-226. DOI: 10.1080/01443615.2016.1188184.
36. Royal College of Obstetricians & Gynaecologists, the Royal College of Anaesthetists. Classification of urgency of caesarean section - a continuum of risk, 2021. Accessed October 15, 2024. <https://www.nice.org.uk/guidance/ng192>.
37. Royal Australian and New Zealand College of Obstetricians and Gynaecologists (RANZCOG). Categorisation of Urgency: Caesarean Section, 2020. Accessed October 15, 2024. <https://ranzcog.edu.au/wp-content/uploads/Categorisation-Urgency-Caesarean-Section.pdf>.
38. World Health Organization. (2016). Quality of care: A process for making strategic choices in health systems. Geneva: WHO. Retrieved from <https://www.who.int/publications/i/item/9789241564652>.
39. National Institute for Health and Care Excellence (NICE). (2019). Quality Standards and Clinical Audit. Retrieved from <https://www.nice.org.uk/standards-and-indicators/quality-standards-and-clinical-audit>.
40. Thaddeus S, Maine D. Too far to walk: Maternal mortality in context. *Social Science & Medicine.* 1994: 38(8), 1091-1110. doi:10.1016/0277-9536(94)90226-
