# Supplementary material for: Caesarean section Robson classification, complications, and lessons learned in a rural hospital in Walikale, North Kivu, Democratic Republic of Congo: a cross-sectional study
Source: AJOG Glob Rep. 2025 Nov 23;6(1):100586. doi: 10.1016/j.xagr.2025.100586 (PMC12771099; doi:10.1016/j.xagr.2025.100586)
Supplement: Supplementary file 3 [file mmc3.docx]

**Additional file 3.** Maternal and perinatal characteristics and outcomes, extensive version

|  | **Total**  n=868 | **Vaginal birth**  n=732 (%) | **CS**  n=136 (%) | **P value** |
| --- | --- | --- | --- | --- |
| **Maternal and pregnancy characteristics** | | | | |
| **Maternal age** (years) | | | | |
| <20 | 200 (23.0) | 178 (24.3) | 22 (16.2) | 0.11 |
| 20 - 34 | 585 (67.4) | 484 (66.1) | 101 (74.3) |  |
| ≥ 35 | 83 (9.6) | 70 (9.6) | 13 (9.6) |  |
| **Distance of village (minutes)** | | | | |
| <30 min | 523 (60.5) | 465 (63.8) | 58 (42.6) | **<0.01** |
| ≥ 30 and < 60 min | 136 (15.7) | 103 (14.2) | 33 (24.3) |  |
| ≥ 60 and <120 min | 94 (10.9) | 71 (9.7) | 23 (16.9) |  |
| ≥ 120 min | 112 (12.9) | 90 (12.3) | 22 (16.2) |  |
| *Missings* | *3 (0.3)* | *3 (0.4)* | *0 (0)* |  |
| **Location of village** | | | | |
| Centre Walikale | 458 (53.0) | 407 (55.9) | 51 (37.5) | **<0.01** |
| Axe Kisangani | 237 (27.3) | 186 (25.5) | 51 (37.5) |  |
| Axe Goma | 82 (9.5) | 65 (8.9) | 17 (12.5) |  |
| Axe Bukavu | 88 (10.2) | 71 (9.7) | 17 (12.5) |  |
| *Missings* | *4 (0.4)* | *4 (0.5)* | *0 (0)* |  |
| **Parity** | | | | |
| Primiparity | 224 (25.8) | 198 (27.0) | 26 (19.1) | 0.19 |
| Low multiparity (P1-5) | 409 (47.2) | 339 (46.3) | 70 (51.5) |  |
| Grand multiparity (P6-9) | 193 (22.2) | 158 (21.6) | 35 (25.7) |  |
| Grand grand multipara (P≥10) | 42 (4.8) | 37 (5.1) | 5 (3.7) |  |
| **Previous CS** | | | | |
| Yes | 191 (22.0) | 102 (13.9) | 89 (65.4) | **<0.01** |
| No | 677 (78.0) | 630 (86.1) | 47 (34.6) |  |
| **Number of previous CS** | | | | |
| One | 111 (12.8) | 79 (10.8) | 32 (23.5) | **<0.01** |
| Two | 46 (5.3) | 23 (3.1) | 23 (16.9) |  |
| Three or more | 34 (3.9) | - | 34 (25.0) |  |
| **Pregnancy interval since previous CS**, n= 191 | | | | |
| <6 months | 4 (2.5) | 1 (1.1) | 3 (4.1) | 0.07 |
| 6 – 17 months | 34 (20.9) | 14 (15.6) | 20 (27.4) |  |
| ≥ 18 months | 125 (76.6) | 75 (83.3) | 50 (68.5) |  |
| *Missings* | *28 (14.7)* | *12 (6.3)* | *16 (8.3)* |  |
| **Antenatal care** | | | | |
| Yes | 765 (99.4) | 649 (99.5) | 116 (98.3) | 0.31 |
| No | 5 (0.6) | 3 (0.5) | 2 (1.7) |  |
| *Missings* | *98 (11.3)* | *80 (10.9)* | *18 (13.2)* |  |
| **Antenatal care no. of consultations**, n=765 | | | | |
| Less than four | 510 (74.3) | 426 (74.0) | 84 (76.4) | 0.34 |
| Four or more | 176 (25.7) | 150 (26.0) | 26 (25.6) |  |
| *Missings* | *421 (48.5)* | *362 (49.5)* | *59 (43.3)* |  |
| **Blood group** | | | | |
| O | 131 (48.9) | 73 (48.3) | 58 (49.6) | 0.79 |
| A | 74 (27.6) | 45 (29.8) | 29 (24.8) |  |
| B | 48 (17.9) | 25 (16.6) | 23 (19.7) |  |
| AB | 15 (5.6) | 8 (5.3) | 7 (6.0) |  |
| *Missings* | *600 (69.1)* | *581 (79.4)* | *19 (14.0)* |  |
| **Rhesus** | | | | |
| Positive | 266 (95.7) | 154 (95.7) | 112 (95.7) | 1.00 |
| Negative | 12 (4.3) | 7 (4.3) | 5 (4.3) |  |
| *Missings* | *590 (68.0)* | *571 (78.0)* | *19 (14.0)* |  |
| **HIV** | | | | |
| Positive | 11 (1.6) | 11 (1.9) | 0 (0) | 0.39 |
| Negative | 683 (98.4) | 577 (98.1) | 106 (100) |  |
| *Missings* | *174 (20.0)* | *144 (19.7)* | *30 (22.1)* |  |
| **Traditional medication used** | 35 (4.0) | 29 (4.0) | 6 (4.4) | 0.81 |
| **Number of foetuses** | | | | |
| Singleton | 851 (98.0) | 724 (98.9) | 127 (93.4) | **<0.01** |
| Twins | 16 (1.9) | 7 (1.0) | 9 (6.6) |  |
| Triplets | 1 (0.1) | 1 (0.1) | 0 (0) |  |
| **Foetal presentation** | | | | |
| Cephalic | 840 (96.8) | 718 (98.1) | 122 (89.7) | **<0.01** |
| Breech | 21 (2.4) | 14 (1.9) | 7 (5.1) |  |
| Transverse | 7 (0.8) | 0 (0) | 7 (5.1) |  |
| **Admission to maternity waiting home** | 24 (2.8) | 18 (2.5) | 6 (4.40) | 0.25 |
| **Childbirth information** | | | | |
| **Gestational age** | | | | |
| Very preterm (< 32 weeks)^1^ | 7 (1.6) | 4 (1.1) | 3 (3.9) | 0.72 |
| Moderate preterm (32 to 33^+6^ weeks) | 10 (2.2) | 8 (2.2) | 2 (2.6) |  |
| Late preterm (34 to 36^+6^ weeks) | 20 (4.5) | 16 (4.3) | 4 (5.2) |  |
| Term (37 to 41^+6^ weeks) | 352 (78.7) | 293 (79.2) | 59 (76.6) |  |
| Post-term (≥ 42 weeks) | 58 (13.0) | 49 (13.2) | 9 (11.7) |  |
| *Missings* | *421 (48.5)* | *362 (49.5)* | *59 (43.2)* |  |
| **Time of birth** | | | | |
| Day shift (8h00 – 15h59) | 266 (30.6) | 228 (31.4) | 38 (27.9) | **<0.01** |
| Evening shift (16h00 – 23h59) | 316 (36.4) | 255 (35.1) | 61 (44.9) |  |
| Night shift (00h00 – 07h59) | 281 (32.4) | 244 (33.6) | 37 (27.2) |  |
| **Vacuum-assisted delivery attempted** | 52 (6.0) | 42 (5.7) | 10 (7.4) | 0.29 |
| **Labour induction** | 17 (2.0) | 12 (1.6) | 5 (3.7) | 0.12 |
| **Oxytocin augmentation** | 35 (4.0) | 26 (3.6) | 9 (6.6) | 0.10 |
| **Episiotomy** | 111 (12.7) | 107 (14.6) | 4 (2.9) | **<0.01** |
| **Active management of the 3^rd^ stage of labour** | 868 (100) | 732 (100) | 136 (100) | 1.00 |
| **Contraception by tubal ligation** | 18 (2.1) | 6 (0.8) | 12 (8.8) | **<0.01** |
| **Perinatal characteristics** | | | | |
| **Sex** | | | | |
| Male | 451 (52.1) | 375 (51.4) | 76 (55.9) | 0.10 |
| Female | 414 (47.9) | 354 (48.6) | 60 (44.1) |  |
| *Missings* | *3 (0.3)* | *3 (0.4)* | *0 (0)* |  |
| **Birth weight** | | | | |
| Very low (<1500 grams)^1^ | 10 (1.2) | 5 (0.7) | 5 (3.7) | **<0.01** |
| Low (1500 – 2499 grams) | 137 (15.8) | 111 (15.2) | 26 (19.1) |  |
| Normal (≥ 2500 grams) | 720 (83.2) | 615 (84.1) | 105 (77.2) |  |
| *Missings* | *1 (0.1)* | *1 (0.1)* | *0 (0)* |  |
| **Apgar 1 minute** (n=840, excluding stillbirths) | | | | |
| Asphyxia (score 0-4) | 28 (3.4) | 17 (2.4) | 11 (8.6) | **<0.01** |
| Difficulty adapting (score 5-7) | 340 (40.7) | 266 (37.6) | 74 (5.8) |  |
| Normal (score 8-10) | 467 (55.9) | 424 (60.0) | 43 (33.6) |  |
| *Missings* | *5 (0.6)* | *5 (0.7)* | *0 (0)* |  |
| **Apgar 5 minutes** (n=840, excluding stillbirths) | | | | |
| Asphyxia (score 1-6) | 51 (6.1) | 29 (4.1) | 22 (17.2) | **<0.01** |
| Difficulty adapting (score 7-8) | 297 (35.6) | 237 (33.5) | 60 (46.9) |  |
| Normal (score 9-10) | 487 (58.3) | 441 (62.4) | 46 (35.9) |  |
| *Missings* | *5 (0.6)* | *5 (0.7)* | *0 (0)* |  |
| **Admission to neonatal unit** | 80 (9.2) | 44 (6.0) | 38 (27.9) | **<0.01** |
| **Duration of admission neonatal unit**, n=73 | | | | |
| Short (< 7 days) | *39 (53.4)* | 670 (97.4) | 82 (83.7) | **<0.01** |
| Long (≥ 7 days) | *34 (46.6)* | 18 (2.6) | 16 (16.3) |  |
| *Missings* | *7 (0.8)* | *3 (0.4)* | *4 (2.9)* |  |
|  |  |  |  |  |
| **Maternal complications** | | | | |
| **Maternal death** | 4 (0.5) | 1 (0.1) | 3 (2.2) | **<0.01** |
| **Maternal near miss** | 35 (4.0) | 6 (0.8) | 29 (21.3) | **<0.01** |
| **Uterine rupture** | 13 (1.5) | 0 (0) | 13 (9.6) | **<0.01** |
| **Eclampsia** | 0 (0) | 0 (0) | 0 (0) | *1.00* |
| **Placental abruption** | 6 (0.7) | 0 (0) | 6 (4.4) | **<0.01** |
| **Abnormally invasive placenta** | 7 (0.8) | 1 (0.1) | 6 (4.4) | **<0.01** |
| **Severe haemorrhage** | 52 (6.0) | 28 (3.8) | 24 (17.6) | **<0.01** |
| **Haemorrhage requiring blood transfusion** | 32 (3.7) | 10 (1.4) | 22 (16.2) | **<0.01** |
| *One or two units of whole blood* | *20 (2.3)* | *8 (1.1)* | *12 (8.8)* |  |
| *Three or more units of whole blood* | *12 (1.4)* | *2 (0.3)* | *10 (7.4)* |  |
| **Maternal sepsis** | 21 (2.4) | 4 (0.5) | 17 (2.5) | **<0.01** |
| **Malaria during childbirth** | 22 (2.5) | 16 (2.1) | 6 (4.4) | *0.14* |
| **Perinatal complications** | | | | |
| **Perinatal death** | 53 (6.1) | 32 (4.4) | 21 (15.4) | **<0.01** |
| **Stillbirth** | 28 (3.2) | 20 (2.8) | 8 (5.9) | *0.07* |
| *Antepartum* | *21 (2.4)* | *14 (1.9)* | *7 (5.1)* |  |
| *Intrapartum* | *7 (0.8)* | *6 (0.8)* | *1 (0.7)* |  |
| **Neonatal death** | 25 (2.9) | 12 (1.6) | 13 (9.6) | **<0.01** |
| *Immediately (during resuscitation)* | *13 (1.5)* | *6 (0.8)* | *7 (5.2)* |  |
| *Early (day 1-7)* | *8 (0.9)* | *4 (0.5)* | *4 (2.9)* |  |
| *Late (day 8-28)* | *4 (0.4)* | *2 (0.3)* | *2 (1.5)* |  |
| **Neonatal near miss** (composite, n=840)  Asphyxia, neonatal infection, sepsis, intraventricular  haemorrhage, respiratory distress (excluding stillbirths) | 76 (9.0) | 45 (6.3) | 31 (24.2) | **<0.01** |
| **Adverse neonatal outcome** (composite)  Neonatal death, admission to neonatal department,  preterm <37 weeks, low birth weight <2500 grams. | 209 (24.1) | 155 (21.2) | 54 (39.7) | **<0.01** |
| **Legend**  ^1^ Four births occurred at 22–28 weeks of gestation with birth weights of 500–1000 grams, despite being non-viable in this setting. Two were emergency cesarean sections—one for placenta previa and one performed perimortem during maternal resuscitation of a woman who died. The other two were vaginal births, resulting in live infants (850 and 950 grams) who survived for several days with kangaroo care and received neonatal care. | | | | |
