# Supplementary material for: Caesarean section Robson classification, complications, and lessons learned in a rural hospital in Walikale, North Kivu, Democratic Republic of Congo: a cross-sectional study
Source: AJOG Glob Rep. 2025 Nov 23;6(1):100586. doi: 10.1016/j.xagr.2025.100586 (PMC12771099; doi:10.1016/j.xagr.2025.100586)

|  |  |  | **Among women with CS**  **(n=136)** | | | **Among the entire obstetric population**  **(n=868)** | | |
| --- | --- | --- | --- | --- | --- | --- | --- | --- |
|  | **No. of CS** | **No. of deliveries** | **Severe PPH** | **Severe infections** | **Perinatal deaths** | **Severe PPH** | **Severe infections** | **Perinatal deaths** |
| **Robson 1**  Nulliparous, with a single cephalic pregnancy, at ≥37 weeks’ gestation, spontaneous labour. | 19 | 198 | 2  (10.5%) | 5  (26.3%) | 2  (10.5%) | 9  (4.5%) | 9  (4.5%) | 8  (4.0%) |
| **Robson 2**  Nulliparous, with a single cephalic pregnancy, at ≥37 weeks’ gestation, induced labour or CS before labour. | 2 | 4 | - | - | - | 1  (25.0%) | 1  (25.0%) | 1  (25.0%) |
| **Robson 3**  Multiparous, without a previous CS scar, with a single cephalic pregnancy at ≥37 weeks’ gestation, spontaneous labour. | 12 | 409 | 1  (8.3%) | 1  (8.3%) | 2  (16.7%) | 15  (3.7%) | 3  (0.7%) | 12  (2.9%) |
| **Robson 4**  Multiparous, without a previous CS scar, a single cephalic pregnancy, at ≥37 weeks’ gestation, induced labour or CS before labour. | 1 | 8 | - | - | - | - | - | 2  (25.0%) |
| **Robson 5**  Multiparous, with at least one CS scar and a single cephalic pregnancy, at ≥37 weeks’ gestation. | 68 | 164 | 18  (26.5%) | 3  (4.4%) | 7  (10.3%) | 20  (29.4%) | 5  (7.4%) | 8  (11.8%) |
| **5.1 -** One previous CS | 22 | 97 | 4  (18.2%) | 2  (9.1%) | 3  (13.6%) | 5  (5.2%) | 3  (3.1%) | 3  (3.1%) |
| **5.2 -** Two previous CS | 19 | 40 | 5  (26.3%) | 1  (5.3%) | 2  (10.5%) | 7  (17.5%) | 1  (2.5%) | 3  (7.5%) |
| **5.3 -** Three or more previous CS | 27 | 27 | 9  (33.3%) | - | 2  (7.4%) | 8  (29.6%) | 1  (3.7%) | 2  (7.4%) |
| **Robson 6**  Nulliparous, with a single breech pregnancy. | 1 | 4 | 1  (100%) | - | - | - | - | - |
| **Robson 7**  Multiparous, with a single breech pregnancy (incl. previous CS scar). | 4 | 14 | 1  (25.0%) | - | - | 2  (14.3%) | - | 3  (21.4%) |
| **Robson 8**  Multiple pregnancies (incl. previous CS scar). | 9 | 17 | 2  (22.2%) | 1  (11.1%) | 3  (33.3%) | 1  (5.9%) | 1  (5.9%) | 7  (41.2%) |
| **Robson 9**  Single pregnancy with transverse or oblique lie (incl. previous CS scar). | 8 | 8 | 1  (12.5%) | - | 2  (25.0%) | 1  (14.3%) | - | 2  (25.0%) |
| **Robson 10**  Single cephalic pregnancy at ≤36 weeks’ gestation (incl. previous CS scar). | 12 | 42 | 5  (41.7%) | 2  (16.7%) | 5  (41.7%) | 3  (7.1%) | 2  (4.8%) | 2  (23.8%) |

**Additional file 4.** Maternal complications and perinatal deaths per Robson group

**Maternal and perinatal deaths and near miss** of CS cases, in % per Robson Group


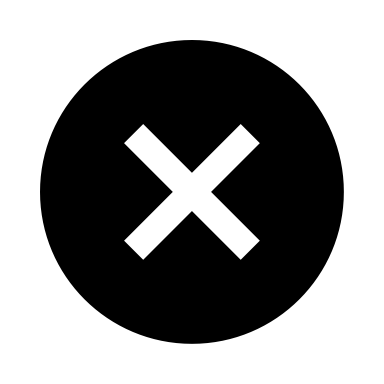


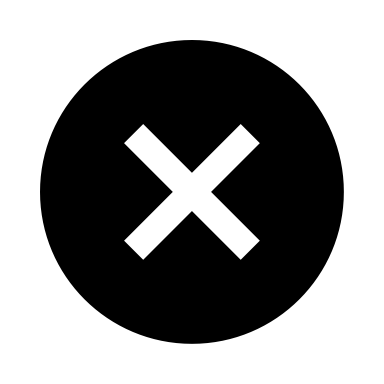

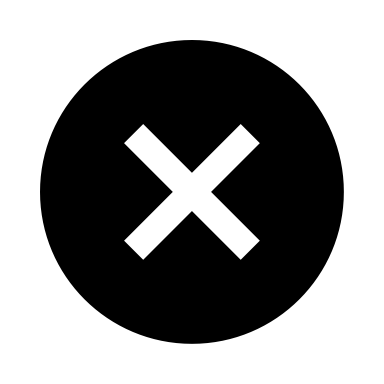

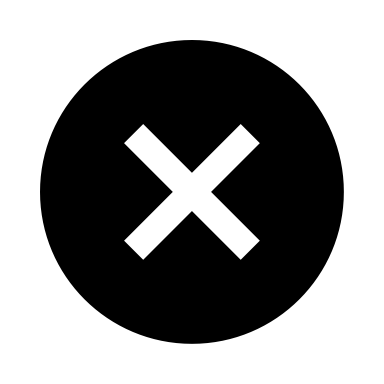

Supplement: Supplementary file 4 [file mmc4.docx]
