# Supplementary material for: Caesarean section Robson classification, complications, and lessons learned in a rural hospital in Walikale, North Kivu, Democratic Republic of Congo: a cross-sectional study
Source: AJOG Glob Rep. 2025 Nov 23;6(1):100586. doi: 10.1016/j.xagr.2025.100586 (PMC12771099; doi:10.1016/j.xagr.2025.100586)
Supplement: Supplementary file 5 [file mmc5.docx]

**Additional file 5.** CS audit outcomes per Robson group

**CS indications absolute vs. relative vs. no indication** according to clinical case review per Robson Group, n=

**CS quality of decision-making** according to clinical case review per Robson Group, n=

**CS level of urgency** according to clinical case review per Robson Group, n=

**Stage of labour at time of CS** per Robson Group, n=
