# Supplementary material for: Caesarean section Robson classification, complications, and lessons learned in a rural hospital in Walikale, North Kivu, Democratic Republic of Congo: a cross-sectional study
Source: AJOG Glob Rep. 2025 Nov 23;6(1):100586. doi: 10.1016/j.xagr.2025.100586 (PMC12771099; doi:10.1016/j.xagr.2025.100586)
Supplement: Supplementary file 6 [file mmc6.docx]

**Additional file 6.** Positive practice and recommendations following clinical case review of all CS

| **Positive practices** | | **Number of cases mentioned** |
| --- | --- | --- |
| **Patient and community factors** | | |
| 1 | Attended antenatal care | 36 |
| 2 | Stayed at maternity waiting home until delivery due to high-risk pregnancy | 12 |
| 3 | Arrival on time, in latent stage of labour, despite living >2 hours from hospital | 10 |
| 4 | Came with consent letter from husband to perform tubal ligation | 8 |
| **Referral system and access to care** | | |
| 1 | Good access to obstetrical care in hospital, free of charge (MSF supported) | 22 |
| 2 | Referral to hospital by health centre before delivery in case of previous CS | 12 |
| 3 | Motor transport was arranged by family within short time span | 8 |
| 4 | Health centre sent patient with a clear referral letter | 5 |
| **Quality of clinical care** | | |
| 1 | Short interval between decision to perform CS and the birth | 77 |
| 2 | Good surveillance of mother and baby and protocols followed | 38 |
| 3 | Availability of doctor around the clock | 25 |
| 4 | Less experienced doctors and nurses ask colleague for help | 15 |
| 5 | Availability and good use of ultrasound enhanced decision making and reduced severity of complications | 10 |
| 6 | Good use of partograph with adequate documentation | 6 |
| 7 | Ventouse performed for foetal distress in expulsion phase | 5 |
| 8 | Women are mobile during latent and active stage of labour, offered food and water, motivated to walk around and urinate spontaneously | 5 |
| 9 | The MSF protocols are in the delivery room and are used daily by doctors and midwives | 4 |
| 10 | The indication for the CS was discussed with the patient and alternative was provided | 4 |

| **Recommendations** | | **No. of cases mentioned** |
| --- | --- | --- |
| **Patient and community factors** | | |
| 1 | Sensibilization about risks of repeated CS and importance of contraception | 48 |
| 2 | Importance of knowing LMP / gestational age to reduce pregnancy risks | 59 |
| 3 | The harmful effects of traditional medication _(Kashisha)_ | 10 |
| 4 | Allowance of birth companion would enhance cooperation and increase vaginal births | 9 |
| 5 | Education about nutritional needs and anaemia prevention during pregnancy | 8 |
| 6 | Importance of knowing obstetric history (and telling the truth), e.g. on the no. of prior CS | 5 |
| 7 | Antenatal visit card should always be brought for delivery | 4 |
| 8 | In case of high-risk pregnancy or home >30 minutes from hospital, it is highly recommended to stay in maternity waiting home or family in city awaiting delivery | 4 |
| **Referral system and access to care** | | |
| 1 | Make antenatal care free of charge and ensure availability of basic medications | 26 |
| 2 | Put in place free ambulance (or motorcycle if not possible) for referral of emergency obstetric cases | 16 |
| 3 | Implement referral criteria in health centre to aid decision-making for referral to maternity waiting home and hospital during pregnancy, labour and postnatal period | 12 |
| 4 | Private clinics need to adhere to obstetric guidelines, with quality monitoring by the government, and collaborate better with referral centres | 8 |
| 5 | Admission of women with pregnancy complications (such as malaria) should always be in a hospital and not in health centre, and they should be referred timely | 6 |
| 6 | Implementation of non-pneumatic anti shock garment for postpartum haemorrhage in health centres can possibly prevent several severe cases | 3 |
| **Quality of clinical care** | | |
| 1 | Clinical supervision by experience obstetrician needs to be made possible for clinical decision-making and management of difficult cases | 55 |
| 2 | Improving quality of ANC (staffing, availability of medication, vaccines, etc) | 39 |
| 3 | Management of prolonged labour and induction of labour needs improvement (with revision of local practice and introduction of new generation partograph) | 33 |
| 4 | Quantify blood loss during childbirth and CS to prevent underestimation and implement local blood bank | 29 |
| 5 | Improvement of foetal monitoring, especially in high-risk cases (with for example introduction of CTG or Moyo-doptone) | 24 |
| 6 | Enable option for referral of patient to higher centre for complicated cases (such as severe wound infections, need for respiratory support, or need for extra diagnostics) | 8 |
| 7 | A list with all absolute indications should be made available and every CS for non-absolute indications should be discussed with senior clinician | 8 |
| 8 | Educational sessions of doctors and midwives to improve decision-making (indications) and technique of vacuum-assisted delivery | 6 |
| 9 | Patients need to be informed better about what happened (e.g. uterine rupture), so if they attend care at another hospital in the future a better risk assessment can be made | 5 |
| 10 | TeleMedecine should be made accessible through a personal account for every senior clinician to discuss difficult cases with medical specialists | 5 |
| 11 | Regular obstetric trainings and audit of cases for whole team to improve basic obstetric care provided | 4 |
| 12 | If patient consented for tubal ligation, but this is not possible during CS due to the many adhesions, consider placing an intra-uterine device instead | 3 |
| 13 | Ensure that an ultrasound is made in every woman with 3 or more previous CS to assess placenta praevia and possibly increta to anticipate difficult CS | 3 |
| 14 | Employ more midwifes and nurses to ensure good monitoring of very sick women and consider making a high-care unit with a dedicated nurse | 2 |
| 15 | Improve communication and collaboration with health care centres when referral is needed, or if information on ANC is needed | 2 |
