# Supplementary material for: Caesarean section Robson classification, complications, and lessons learned in a rural hospital in Walikale, North Kivu, Democratic Republic of Congo: a cross-sectional study
Source: AJOG Glob Rep. 2025 Nov 23;6(1):100586. doi: 10.1016/j.xagr.2025.100586 (PMC12771099; doi:10.1016/j.xagr.2025.100586)
Supplement: Supplementary file 7 [file mmc7.pdf]

Visual abstract

Cross-sectional study & clinical audit

868 births; 136 caesarean sections (CS)

C-SECTION STUDY

in Walikale, Nord Kivu, DR Congo in 2024

200-bed Rural Referral Hospital

CS rate **16%**

General Referral Hospital Walikale and MEDECINS SANS FRONTIERES

CS → **Who?**

of all CS
50% Robson-5
14% Robson-1

Why?

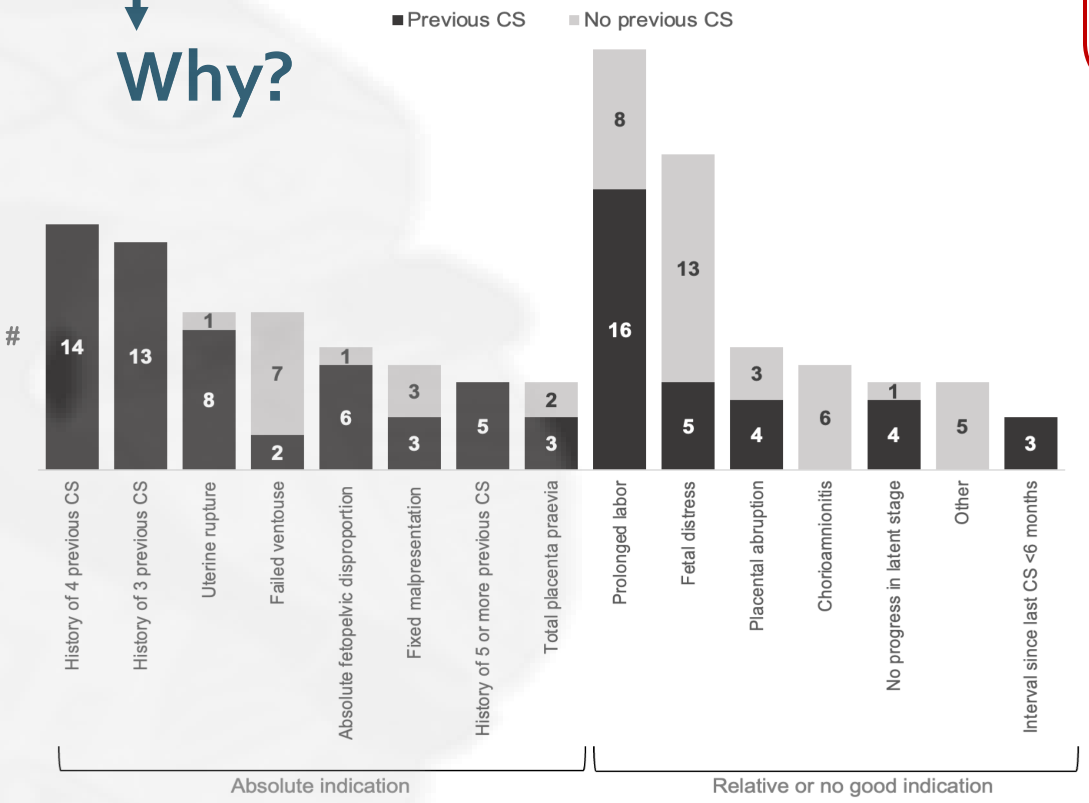

1 in 3 women have a severe complication

1 in 5 women giving birth have a previous CS scar

Which means: very high-risk population

1 in 4 of CS are conducted <u>too early</u> (implies overuse)

Improve access to contraception

Enhance clinical supervision

Ensure an ambulance service

Essential to implement local recommendations to improve safety of birth

Need to elaborate Robson with indication-based matrix and quality-of-care indicators

Need for context-tailored guidelines on CS indications and CS-decision-making for low-resource settings

Key Findings

C-SECTION STUDY

in Walikale, Nord Kivu, DR Congo

In 2024

GENERAL REFERRAL HOSPITAL WALIKALE

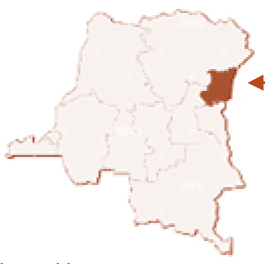

supported by MEDECINS SANS FRONTIERES

conducted by

G. Bushu, C. Kambale, S. Kikwabantu, F. Boelongo, W. Weber, B. Black, K. Verschueren

The aim of this **study** was to report the **caesarean section (CS) rate**, the **CS indications**, **maternal and perinatal complications** and assess **quality of care**, with its recommendations serving as a springboard for **improvements and strategy development** both **locally and internationally**.

Methods

Cross-sectional study

of all births with **clinical audit** of all **CS**

in the **Referral Hospital of Walikale**

from January – March 2024

- +/- 200.000 inhabitants
- 1 hospital & 18 primary health centers
- +/- 4500 hospital births/yr
- Fertility rate +/- 6 per woman
- MSF supported since 2012
- Politically unstable
- Poor infrastructure
- & no referral options

Comprehensive overview

Hospital CS rate

16%

n=136/868

1. 50% **Robson Group 5** (previous CS scar, cephalic, term singleton)
2. 14% **Robson Group 1** (nulliparous, cephalic, term singleton)

65% of all CS are in women with a **previous scar**, of whom 2/6 (icon) **≥ 3 prior CS**

30% of all CS are with **severe complications**

- severe hemorrhage requiring **blood transfusion**
- severe deep **wound infections**
- two **maternal deaths** during CS due to placenta increta and hemorrhage

22% of all births are in women with **a previous scar**

who have a risk of
6% uterine rupture
4% abnormally invasive placenta

with **VBAC success** [vaginal birth after CS]

- 71 % after **one** prior CS
- 50 % after **two** prior CS

High perinatal death rate
60/1000 births

2X higher in CS than in vaginal births

Clinical Audit

Clinical decision-making

Good decision | Too early | Too late

Indication of CS

Absolute (life-saving for mother) | Relative (all fetal & non-life saving indications) | None

★★★

- Short decision-delivery interval
- High ANC attendance
- Free hospital care

★☆☆

- Contraception access and use
- Clinical supervision
- Transportation / 2nd delay

What is the Problem?

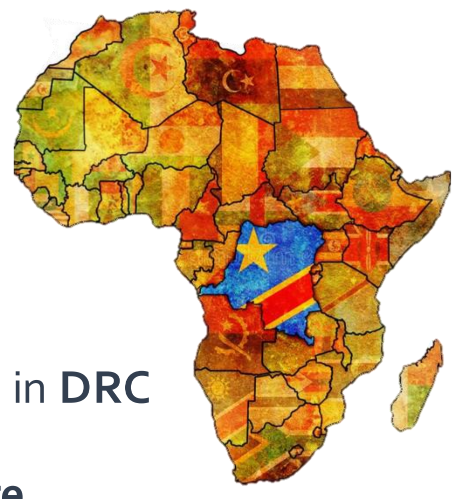

70% of **maternal deaths** occur in **Sub-Sahara Africa** with the most number of deaths in **DRC**

70% in DRC has no or limited **access to care** & the **population-based CS rate** is only **5% in DRC** in 2019

The WHO established an **ideal population-based CS rate** of **10-15%**

However

The risk of ↑ CS access → ↑ CS overuse (poor indications) → ↑ Maternal & perinatal deaths

due to

often **inexperienced doctors** responsible for CS decision-making, with **indications** derived from **research/guidelines in high-income settings**, in a setting with **high peri and postoperative risks and high risk of complications** in **subsequent pregnancies** due to high fertility rates

CS-decision making in **low-resource countries** is very **complex**, but extremely **important**

Globally, better understanding is needed of the **clinical reality** and **challenges** concerning **CS in low-resource settings**

What Our findings imply?

- The **CS rate** seems adequate
- The **VBAC success rate** is high and exemplary
- The **obstetric population** is **complex** and **high risk** due to many women with prior CS (highest in literature)
- The **complication rate** is **high** with more **uterine ruptures** and **abnormally invasive placentas** than ever reported in literature.
- Quality improvement in **CS decision-making** can reduce unnecessary CS (esp. Robson-1) and improve outcomes

What to do?

- Locally **implement** the clinical audit **recommendations** made
- Perform **CS audit** periodically & add quality of care indicators to Robson
- Demand the WHO to develop context-tailored guidelines on CS indications to improve CS-decision-making in low-resource settings

Hôpital de Référence Rural
200 lits

Résumé visuel

Étude transversale et audit clinique

868 accouchements ; 136 césariennes (CS)

Hôpital Géneral de Référence Walikale et MEDECINS SANS FRONTIERES

CESARIENNE ÉTUDE

à Walikale, Nord Kivu, RD Congo dans 2024

Taux de CS **16%**

CS → QUI?

50% Robson-5
14% Robson-1

Pourquoi?

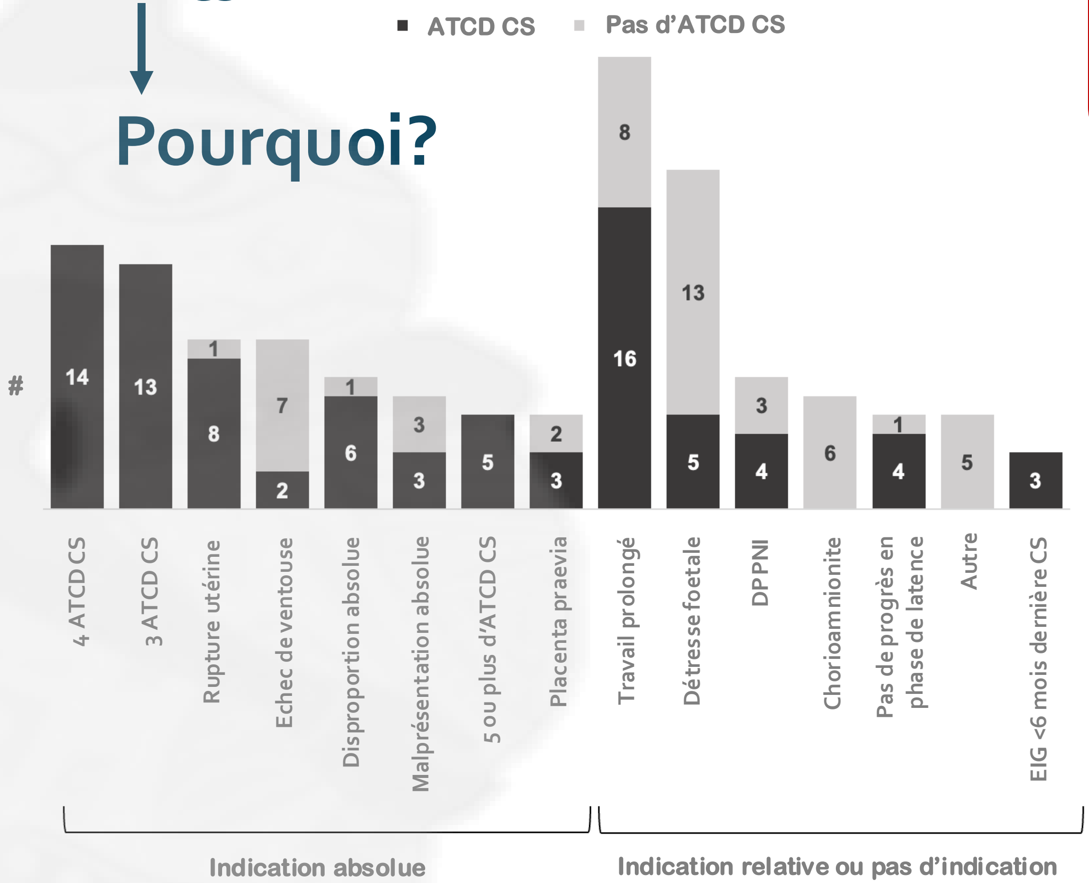

1 : 3 une complication grave du CS

1 : 5 de toutes les femmes ont une cicatrice de césarienne

Ce qui signifie : population à très haut risque

1 : 4 des CS fait trop tôt (implique une surutilisation)

Améliorer l'accès et l'utilisation de la contraception

Améliorer la supervision clinique

Assurer un service d'ambulance

Mise en œuvre des recommandations locales pour améliorer la qualité des soins

L'élaboration de Robson avec une métrique basée sur les indications et des indicateurs de qualité des soins serait utile

Des lignes directrices adaptées au contexte sur les indications de césarienne et la prise de décision en la matière sont absolument nécessaires dans les milieux à faibles ressources

Principales conclusions

ÉTUDE DES CÉSARIENNES

à Walikale, Nord Kivu, RD Congo en 2024

HÔPITAL GÉNERAL DE RÉFÉRENCE WALIKALE

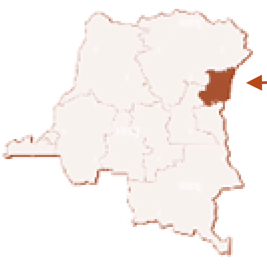

Soutenu par MEDECINS SANS FRONTIERES

par
G. Bushu, C. Kambale, S. Kikwabantu, F. Boelongo, W. Weber, B. Black, K. Verschueren

L'objectif de cette étude était de rapporter **le taux de césarienne** (CS), les **indications** de la CS, les **complications maternelles et périnatales** et **d'évaluer la qualité des soins.** Ses recommandations servent à **améliorer et développer des stratégies** tant au niveau local qu'international.

Méthodes

Étude transversale de toutes les accouchements avec **audit clinique** de toutes les **CS** à l'**hôpital de référence de Walikale** de janvier à mars 2024

+/- 200.000 habitants

1 hôpital et 18 centres de santé

+/- 4500 accouchements/an

Taux de fécondité +/- 6 par femme

MSF soutien depuis 2012

Politiquement instable

Infrastructures déficientes

& aucune option de référence

Quel est le problème ?

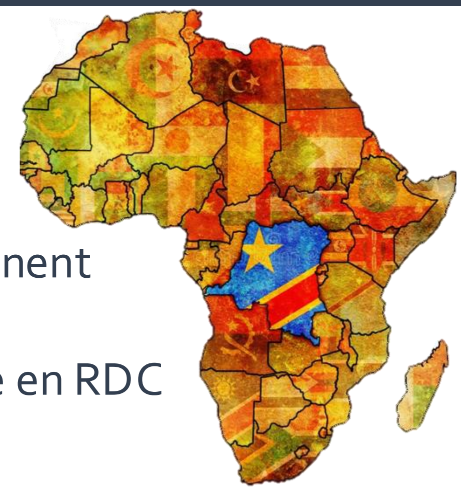

70% des **décès maternels** surviennent en **Afrique subsaharienne** avec le plus grand nombre en RDC

70% en DRC n'a pas ou peu **accès aux soins**

le **taux de CS** (population) est seulement **5%** en DRC en 2019

L'OMS a établi le **taux idéal de CS** de la population de **10-15%**

Cependant

Le risque d'une — Accès à CS → CS surutilisation (faible indications) → Décès maternels et périnatals

en raison de médecins souvent inexpérimentés responsables de la **prise de décision** de la CS, avec des **indications** dérivées de recherches/lignes directrices des pays à revenu élevé, dans un **contexte de risques péri et postopératoires élevés** et de **risque élevé de complications** lors des **grossesses ultérieures** en raison de **taux de fertilité** élevés

La **prise de décision de la CS** est très **complexe**, mais extrêmement **importante** dans les **pays à faibles ressources**

À l'échelle mondiale, une **meilleure compréhension** de la **réalité clinique et des défis liés à la CS** est nécessaire.

Aperçu complet

Taux de CS à l'hôpital

16%

n=136/868

1. 50% — **Robson Group 5** (antécédent cicatrice, céphalique, terme)
2. 14% — **Robson Group 1** (nulliparous, céphalique, terme)

65% de tous les CS sont chez les femmes avec une cicatrice de qui ≥ 3 ATCD CS

30% de tous les CS ont des **complications graves**

- hémorragie grave nécessitant une **transfusion sanguine**
- **infections** des plaies graves/profondes
- deux **décès maternels** pendant la CS en raison d'une hémorragie (placenta increta)

22% de tous les accouchements sont chez les femmes avec une cicatrice qui ont un **risque** de **6 % de rupture utérine** **4 % de placenta anormalement invasif**

avec **Succès de l'AVAC** [accouchement par voie basse après césarienne]

71 % après 1 ATCD CS

50 % après 2 ATCD CS

Taux de mortalité périnatale 60/1000 naissances

2X plus élevé dans les CS que dans accouchements par voie basse

Audit clinique

Prise de décision clinique

Bon décision — Trop tôt — Trop tard

Indication of CS

Absolue (sauvetage pour la mère) — Relative (toutes les indications fœtales et non vitales) — Non

★★★

- **Délai de décision-naissance court**
- **Fréquentation de soins prénatals**
- **Soins gratuits à l'hôpital**

★☆☆

- **Accès de la contraception**
- **Supervision clinique**
- **Transport / 2e délai**

Que signifient nos résultats ?

- Le **taux de CS** semble adéquat; le **taux de l'AVAC** est **élevé et exemplaire**
  - La **population** est **complexe et à haut risque** (beaucoup avec ATCD CS; le plus élevé dans la littérature)
- **L'amélioration de la qualité** dans la prise de décision **en CS** peut réduire les CS inutiles (en particulier Robson-1) et la mortalité maternelle et périnatale

Ce qu'il faut faire?

Mettre en œuvre localement les **recommandations d'audit clinique** formulées

Effectuer des audits CS cliniques réguliers

& ajouter des **indicateurs de qualité** des soins **à Robson**

Exiger que **l'OMS** élabore des **lignes directrices adaptées au contexte** sur les **indications de la CS** afin **d'améliorer la prise de décision** dans les pays à faibles ressources
